# A review of remote sensing-based crop yield estimation: machine learning techniques and environmental, algorithmic, and hardware limitations

**DOI:** 10.3389/fpls.2026.1742689

**Published:** 2026-03-19

**Authors:** Aman Muhammad, Abdul Sattar Mashori, Mansoor Jan, Ziqi Han, Fuzhong Li, Sanaullah Jan, Syed Aziz Shah

**Affiliations:** 1 College of Agricultural Engineering, Shanxi Agricultural University, Jinzhong, China; 2 School of Software, Shanxi Agricultural University, Jinzhong, China; 3 Department of Signal Theory and Communications, Universidad Carlos III de Madrid, Madrid, Spain; 4 School of Computing, Engineering and the Built Environment Edinburgh Napier University, Edinburgh, United Kingdom; 5 Centre for Intelligent Healthcare, Coventry University, Coventry, United Kingdom

**Keywords:** crop productivity prediction and assessment, limitations in yield estimation, machine learning based yield estimation, remote sensing, wireless sensor networks based agriculture

## Abstract

Advancements in agricultural technologies have increasingly emphasized technical innovations aimed at improving the predictability and reliability of agricultural outputs. These aspects encompass developments in agricultural machinery, automation technologies, biotechnology, and controlled environment farming systems. This article focuses on Remote Sensing (RS)-based approaches applied to agricultural yield estimation for both crops and plants. RS technologies offer enhanced precision and scalability, making them particularly effective for large-scale agricultural monitoring and analysis. A systematic classification of RS-based methodologies employed for crop yield estimation is presented in this study. These methodologies are categorized into: (i) Sensor-Based approaches, (ii) Platform-Based approaches, (iii) Analytical and Modeling-based methods, and (iv) Machine Learning (ML)-driven models. Based on findings reported across multiple studies, it is observed that Deep Learning (DL)-based architectures consistently achieve superior performance across key evaluation metrics, including accuracy, precision, recall, and F1-score. This performance advantage stems from their capacity to learn hierarchical representations, capture complex non-linear relationships, scale efficiently with large datasets, and reduce reliance on manual feature engineering. Following this classification, our article presents a comprehensive discussion of the limitations associated with these methodologies. These challenges are organized into four major categories: (i) Environmental, (ii) Algorithmic, (iii) Hardware and Operational, and (iv) Wireless Sensor Networks (WSNs) related limitations. The adopted classification framework helps readers identify and address the key challenges associated with effective yield estimation in crops and plants. Moreover, the article concludes by outlining several future research directions intended to support and guide both early-career and experienced researchers in this domain.

## 1 Introduction

With the integration of technologies such as Remote Sensing (RS), Wireless Sensor Networks (WSNs), and data-driven Machine Learning (ML) modeling approaches, agriculture has entered a new era of enhanced accuracy and precision. These developments make it possible to predict the yield properly and enjoy a greater level of control over agricultural resources (Ji et al., 2021; Shafi et al., 2023). Traditional yield estimation methods have long been employed in conventional farming systems. However, their applicability to modern precision agriculture remains limited. Common practices such as crop cutting experiments, quadrat-based sampling, farmer recall, visual assessment, and plant population counting are predominantly manual and labor-intensive, typically conducted on cereal and field crops (e.g., wheat, rice, and maize) using fixed area quadrats or plot level sampling units (Cheng et al., 2022; Li et al., 2023). These approaches rely on localized measurements taken at discrete spatial scales, which restrict their ability to capture within-field variability and reduce their scalability for large or heterogeneous agricultural landscapes.

Such activities are prone to sampling errors when conducted manually, as data collected from limited sections of a field often fail to accurately represent the overall field conditions (Boros et al., 2024; Valente et al., 2024). These inaccuracies are further exacerbated when crop growth is spatially non-uniform across the field. Moreover, factors such as pest infestations and localized climatic variations introduce additional distortions, leading to increasingly unreliable yield estimates. Error during data collection can substantially affect the accuracy of yield estimation because traditional methods rely on subjective experience rather than objective quantification (Goel et al., 2021; Kosmowski et al., 2021). Traditional approaches may also be inadequate for supporting timely interventions during critical crop growth stages. Although post-harvest weighing provides high accuracy, it is inherently retrospective and lacks proactive decision-making capability, limiting its effectiveness for input optimization in yield estimation (Naik et al., 2022; Pirzada et al., 2023; Shee et al., 2023).

With the advent of RS technologies, agriculture has entered a new era of innovations. Their application has completely transformed traditional agricultural approaches. This transformation has led to a major shift toward data-driven and precision-based farming. It is now widely used for yield estimation in crops and plants. This data-driven approach is primarily based on multispectral, hyperspectral, Light Detection and Ranging (LiDAR), and thermal imaging sensors (Abreu Júnior et al., 2022; Vaidya et al., 2022; Wu et al., 2025). For monitoring reasons, these sensors can be installed on ground-based platforms, satellites, and Unmanned Aerial Vehicles (UAVs). They can capture spectral characteristics that are associated with chlorophyll levels, canopy cover, water stress levels, and nutrient levels. The captured characteristics allow tracking of crop yield potential in a non-destructive and continuous manner. Researchers are increasingly applying ML, Deep Learning (DL), and Artificial Intelligence (AI) to improve the accuracy of predictive models (Ilyas et al., 2023; Morales and Villalobos, 2023; Jabed and Murad, 2024). This advancement enables researchers to extract characteristics efficiently and process large volumes of RS data at once. Some scholars focus on sensor fusion and time-series analysis to achieve deeper temporal understanding and capture dynamic patterns over time. Yield estimation was further enhanced by integrating RS together with Internet of Things (IoT) based on WSNs, which provides real-time decision making (Majumdar et al., 2021; Mowla et al., 2023). This approach enhances sustainability and climate-resilient practices while supporting more accurate prediction of crop and plant yields. Consequently, RS technologies can be regarded as a transformative force in optimizing agricultural productivity and strengthening global food security.

Despite the growing body of review literature on RS-based crop yield estimation, existing studies remain fragmented in terms of technological scope, analytical framing, and methodological limitations. Prior reviews have typically focused on narrow technology subsets, restricted geographic regions, or isolated algorithmic perspectives, which limits their utility for cross-comparison and system-level understanding. For example, Yuan et al. (2024) primarily examined UAV-based yield estimation using ML techniques for selected major crops, while Vaidya et al. (2022) emphasized region-specific datasets from India with a strong reliance on hyperspectral imagery. Similarly, Jabed and Murad (2024) provided a concise overview of yield estimation technologies, but without a detailed analytical or methodological decomposition. Although these studies broadly align with the research direction addressed in this work, none of the existing review articles provide a comprehensive categorization of RS methodologies. Moreover, the aforementioned reviews do not provide a structured, in-depth classification of limitations, such as environmental, algorithmic, hardware-related, and WSNs-based challenges, which this work seeks to organize in a structured manner. In addition, the manuscript surveys commonly adopted ML approaches within RS-driven yield estimation frameworks, outlining their methodological characteristics and areas of application without assuming performance superiority. Furthermore, it consolidates and critically synthesizes the principal challenges affecting RS-based yield estimation systems, including environmental variability, data quality and availability issues, scalability concerns in algorithmic design, hardware and operational constraints, and communication bottlenecks within sensing infrastructures.

The remainder of the paper is structured as follows. Section 2 presents a comprehensive discussion of RS-based yield estimation approaches, which are systematically classified into four subcategories: (i) sensor-based, (ii) platform-based, (iii) analytical modeling-based, and (iv) ML-based methods. This is followed by a dedicated Section 3 that critically examines the limitations encountered by RS-based yield estimation systems. To ensure comprehensive coverage, these limitations are categorized into four major groups: (i) environmental, (ii) algorithmic, (iii) hardware and operational, and (iv) wireless sensor networks related constraints. Section 4 synthesizes and compares the results reported by various researchers, grouping them into meaningful categories to enable readers to clearly assess the relative performance of different ML algorithms. Finally, in the conclusion section, the first paragraph summarizes the overall findings of the study, and the second paragraph outlines prospective future research directions for the active research communities working in this domain.

### 1.1 Research methodology

**Figure 1:**
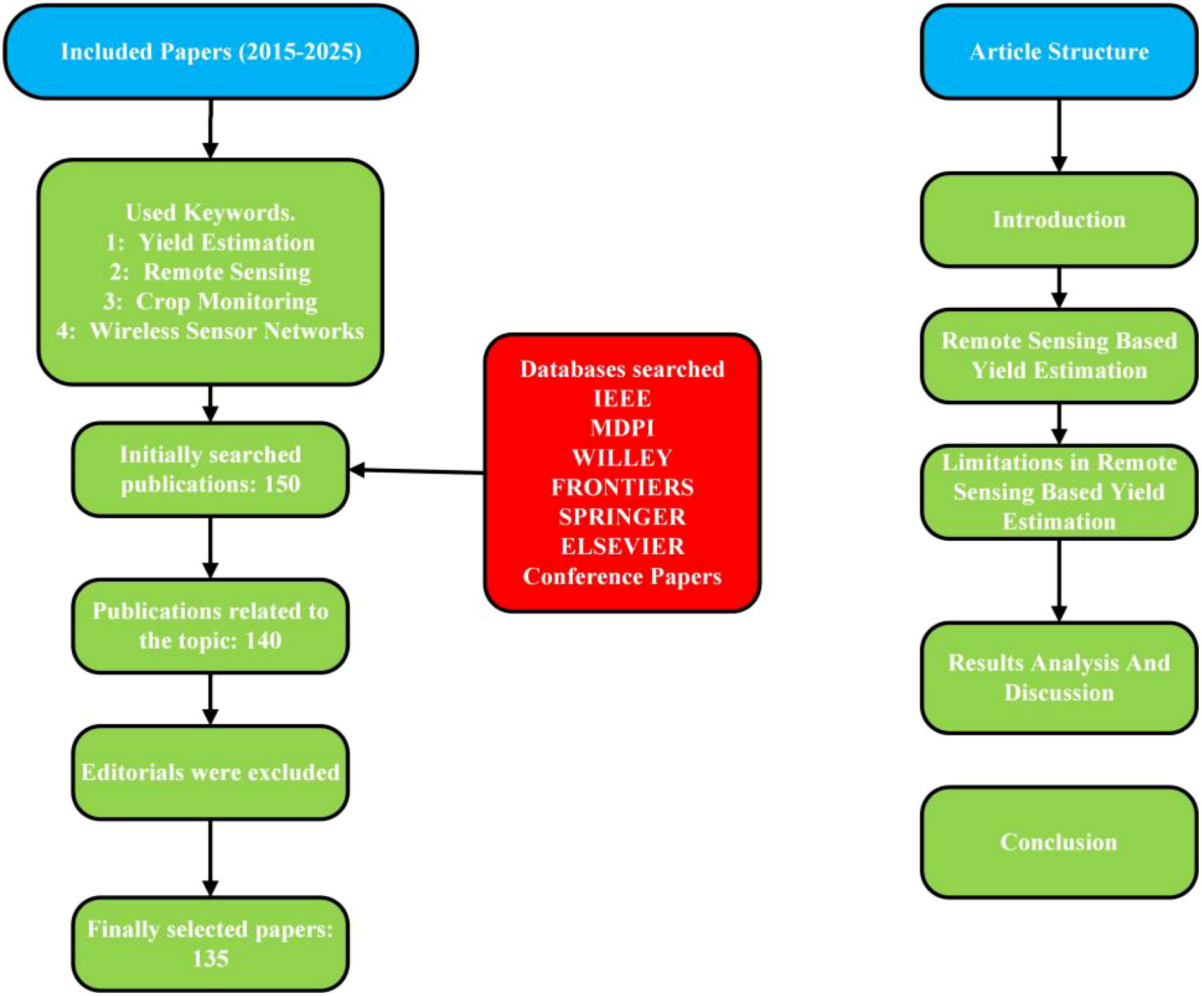
Flowchart of this article and its research methodology.

**Figure 1** illustrates the systematic methodology adopted for conducting this review. It covers studies published between 2015 and 2025. A structured keyword strategy, including yield estimation, RS, crop monitoring, and WSNs were employed to retrieve relevant literature. Multiple reputable scientific databases, such as IEEE, MDPI, Wiley, Frontiers, Springer, and Elsevier, as well as conference proceedings, were comprehensively searched. An initial pool of 150 publications was identified through this process. After relevant screening, 140 studies were found to be closely aligned with the scope of the review. Editorials were excluded to maintain scientific rigor. Following quality assessment and filtering, 135 papers were finally selected for detailed analysis.

## 2 Remote sensing-based yield estimation

**Figure 2:**
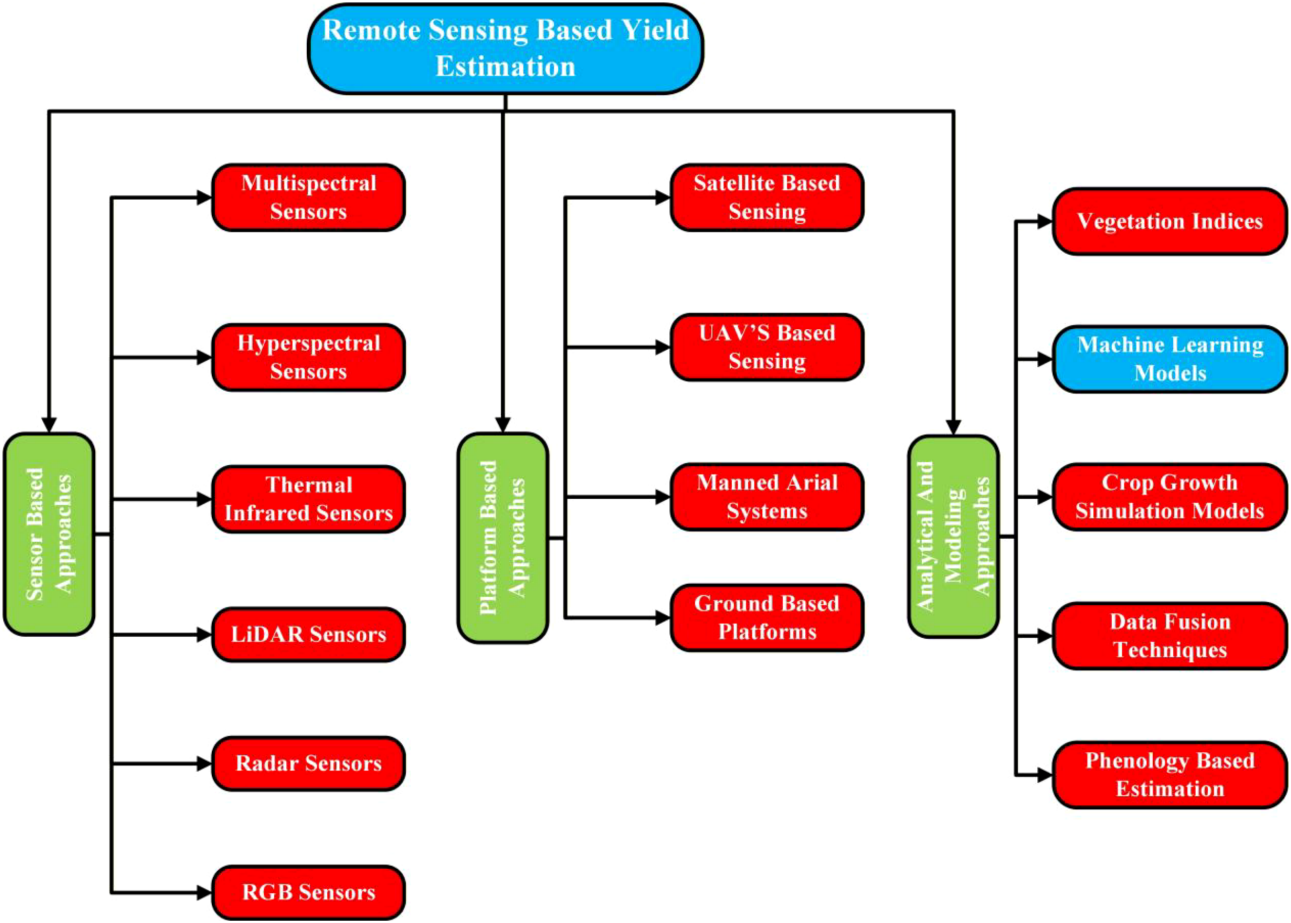
Categorization of remote sensing-based yield estimation.

RS-based yield estimation constitutes a core section of this review, as it establishes the methodological foundation for understanding how spatial, spectral, and temporal information can be systematically exploited to quantify crop productivity. The primary purpose of this section is to synthesize and contextualize existing approaches that utilize satellite, UAVs, and proximal sensing data for yield prediction, while highlighting their operational principles and analytical assumptions. By outlining sensor modalities, data acquisition strategies, and modeling paradigms, this section provides a structured basis for critically evaluating model performance, scalability, and robustness (Falcioni et al., 2023; Asner et al., 2024). To improve clarity for the reader, we have categorized this section into three groups: (i) Sensor-Based Approaches, (ii) Platform-Based Approaches, and (iii) Analytical and Modeling-based Approaches. **Figure 2** is provided as a flowchart that elaborates the categorization and sub-categorization of the RS-based yield estimation methodologies in crops and plants.

### 2.1 Sensor-based approaches

Sensor-based techniques for yield estimation in plants employ advanced sensing tools that directly monitor vital biophysical and biochemical parameters. Devices such as multispectral, hyperspectral, LiDAR, and thermal sensors generate critical data on canopy characteristics, chlorophyll concentration, and stress conditions, enabling accurate, real-time yield forecasting and enhanced farm management.

#### 2.1.1 Multispectral sensors

The use of multispectral sensors is an essential technological advancement in precision agriculture because it has the ability to provide quantitative measurements of crop physiological characteristics through vegetation indices, such as the Normalized Difference Vegetation Index (NDVI) and the Enhanced Vegetation Index (EVI). These sensors record the surface reflectance across specific spectral intervals, mainly within the visible spectrum (400 to 700 nm) and the near-infrared spectrum (700 to1300 nm). The NDVI utilizes the difference between red and near-infrared reflectance to evaluate chlorophyll content and photosynthetic activity, whereas the EVI incorporates correction factors that may reduce atmospheric noise and canopy background influences, delivering higher precision and sensitivity under dense vegetation and high-biomass conditions (Son et al., 2014; Abreu Júnior et al., 2022). Some researchers have also suggested that integrating this technology with UAV- or satellite-based imaging systems enables multispectral sensors to support high-resolution, spatiotemporal monitoring of broad agricultural landscapes in a repeatable and non-destructive way (Sunoj et al., 2021).

#### 2.1.2 Hyperspectral sensors

The adoption of hyperspectral sensors is increasing in precision agriculture, primarily due to their capability to capture data with extremely fine spectral resolution. This characteristic of hyperspectral sensors spans hundreds of narrow wavelength bands, thereby enabling a highly detailed evaluation of the biochemical and physiological conditions of crops. Data acquired from these narrow spectral bands enable hyperspectral imaging to identify subtle variations in leaf pigments, plant water content, and even soil properties. This information facilitates the early detection of plant stress signatures at both the molecular and physiological scales, thereby supporting timely intervention strategies. It further enables the precise quantification of critical agronomic indicators, including chlorophyll concentration, carotenoid ratios, canopy biochemical composition, and photosynthetic efficiency parameters (Vaidya et al., 2022). Hyperspectral data further support the generation of rich, multidimensional spectral profiles of vegetation. When these profiles are analyzed using radiative transfer models, chemometric approaches, and ML techniques, they provide enhanced insight into vegetation properties and conditions, enabling robust and reliable predictions of biomass accumulation, crop vitality, and overall yield. Zhang et al. (2023) applied Hyperspectral RS technology for tobacco quality estimation, yield prediction, and stress detection. Their findings indicate that integrating physiological trait detection with spatiotemporal crop growth simulations positions hyperspectral sensing as a transformative technology in precision agriculture due to its ability to deliver near real-time insights.

#### 2.1.3 Thermal infrared sensors

Thermal infrared (TIR) sensors are an integrally important technology that researchers have recently started to use in precision agriculture. This camera-based technology provides researchers with accurate measurements of canopy temperature. Those measurements will act as a dependable indicator of plant water status and transpiration processes. However, the variations in canopy temperature can also be associated with stomatal behavior and evaporative cooling, serving as an early signal of water stress that can impair photosynthetic efficiency, carbon fixation, and biomass production. A widely adopted technique for evaluating such stress is the Crop Water Stress Index (CWSI), which is formulated as Equation 1.

(1) $$CWSI=\frac{T_{C}-T_{WET}}{T_{DRY}-T_{WET}}$$

Here, $T_{C}$ denotes the observed canopy temperature, $T_{WET}$ represents the temperature of a fully transpiring well-watered canopy, and $T_{DRY}$ stands for temperature of a non-transpiring severely stressed canopy (Katimbo et al., 2022). Shen et al. (2022a) employed a hybrid sensing framework combining TIR and hyperspectral imaging, with wheat as the crop of interest in their analysis. They aimed to improve the yield estimation through this hybrid sensor-based approach and reported that incorporating TIR-based temperature data with vegetation indices and crop growth models enables precise spatiotemporal assessment of irrigation needs, stress dynamics, and yield projections in crops and plants. Thus, it can be inferred that TIR sensing provides a non-invasive, scalable, and scientifically robust approach to linking plant stress physiology with predictive yield estimation across various agricultural systems.

#### 2.1.4 LiDAR sensors

LiDAR represents a cutting-edge RS technique for crop and plant yield estimation. This technology helps in generating high resolution Three-Dimensional (3D) point clouds that capture canopy structural characteristics. The structural features captured can be used in determining canopy height, complexity, and volume of biomass (Debnath et al., 2023). LiDAR datasets offer valuable insights into above-ground biomass dynamics, where the resulting model outputs can be utilized to establish a strong relationship with the yield potential of vegetation. Compared to passive optical sensors, LiDAR technology can penetrate complex canopy layers and provide in-depth detail. The obtained information can be combined with radiative transfer modeling, allometric correlations, and ML. Such approaches provide improved yield estimation compared to methods based solely on biophysical metrics (Rivera et al., 2023).

#### 2.1.5 Radar SAR sensors

One of the emerging active microwave RS systems gaining popularity for quantitative yield estimation in agriculture is Synthetic Aperture Radar (SAR). SAR technology can penetrate cloud cover, assisting in creating high-resolution backscatter data. The collected information is highly sensitive to dielectric vegetation and heterogeneity. Radar-based acquired data are variable in nature and can be analyzed to measure soil moisture status and crop phenology (Cunha et al., 2024). Being sensitive to surface roughness and volumetric scattering, SAR enables detection of important biophysical parameters, including aboveground biomass and Leaf Area Index (LAI), Both of which are regarded as critical for estimating yield potential in crops and plants. Moreover, incorporating polarimetric decomposition in SAR further enhances the discriminative capability of the extracted features. These enriched feature representations enable more effective integration with advanced ML approaches, thereby supporting the development of more accurate predictive yield estimation models. These innovations are advantageous for both researchers and farmers, facilitating real-time and non-real-time yield estimation strategies (Ghosh et al., 2022a).

#### 2.1.6 RGB camera sensors

The Red, Green, and Blue (RGB) technology uses visible spectrum reflectance of high resolution in capturing the phenotypic features related to the productivity of plants. These measurements include canopy color differences, derived from indices of red and green intensity ratios. These ratios provide precise data on chlorophyll levels and stress-induced discolorations due to leaf age (Zeng et al., 2021). Researchers have observed that the collected data closely correlate with the performance of photosynthesis, which directly influences biomass accumulation dynamics and contributes to structural variability within the crop canopy. These observations make RGB a reliable indicator of crop and plant production potential. When these RGB-based analyses are coupled with computer vision, feature extraction, and ML algorithms, they enable the development of cost-efficient, scalable, and scientifically robust yield prediction frameworks (Fu et al., 2021).

**Table 1** summarizes the sensor types most frequently and commonly used for yield estimation, while also providing a concise overview of their key applications.

**Table 1:** Sensor types used in agriculture for crops and plants yield estimation.

S.No	Sensor type	Examples	Key applications in yield estimation
1	Optical Sensors	RGB Cameras, Multispectral, Hyperspectral	Canopy cover and structure analysis. Vegetation indices (NDVI, EVI, etc.). Chlorophyll and nutrient status. Crop classification and stress detection.
2	Thermal Sensors	Infrared Thermal Cameras	Canopy temperature monitoring. Water stress detection. Evapotranspiration estimation. Indirect yield prediction under drought.
3	LiDAR Sensors	Airborne or UAV mounted LiDAR	3D canopy structure. Plant height and volume estimation. Biomass assessment. Field variability mapping.
4	Fluorescence Sensors	Chlorophyll Fluorescence Sensors, Pulse Amplitude Modulation (PAM) Fluorometer	Photosynthetic activity measurement. Early stress detection. Crop growth monitoring.
5	Radar/Microwave Sensors	SAR, Passive Microwave Radiometers	Soil moisture estimation. Biomass and crop density measurement. Monitoring under cloud cover or at night. Large scale yield estimation.
6	Proximal/Ground Based Sensors	Spectroradiometers, Soil Plant Analysis Development (SPAD) Meters, Soil Moisture Sensors	Leaf level reflectance and pigment analysis. Chlorophyll concentration measurement. Soil moisture status. Ground truthing for satellite/UAV data.

### 2.2 Platform-based approaches

Platform-based approaches for yield estimation in crops and plants utilize diverse carriers including, (i) satellites, (ii) UAVs, (iii) manned aerial systems and (iv) ground-based platforms. These platforms enable scalable, spatiotemporal monitoring of crop traits and can be equipped with technologies that integrate multisource data for precise, non-destructive assessment of growth dynamics and yield potential.

#### 2.2.1 Satellite-based sensing

Satellite-based RS measurements are essential for determining yield in agricultural applications. This method offers strong multitemporal capabilities because satellite-based orbital data naturally provide consistent and repeatable temporal observations. Many research groups worldwide use these observations-based datasets to analyze crop phenological development and canopy biophysical properties (Lungu et al., 2020). However, researchers have also identified several constraints associated with this technology, most notably its pronounced sensitivity to variable weather conditions. These effects become particularly evident in applications such as soil moisture characterization and the assessment of canopy structural complexity, where fluctuations in atmospheric conditions can substantially influence data reliability and consistency. To mitigate these limitations, several organizations have begun exploring hybrid system architectures that integrate SAR and thermal imagery-based platforms with conventional satellite measurements. Findings indicate that such hybrid approaches enable more reliable capture of canopy temperature signatures, which serve as effective proxies for plant water status and transpiration dynamics. Furthermore, it was reported that the resulting multimodal datasets can be leveraged to train and enhance statistical yield-prediction models with improved robustness and accuracy. As a result, real-time scalability and decision-support systems in precision agriculture become feasible (Vallentin et al., 2022).

#### 2.2.2 UAV-based sensing

RS-based drone yield estimation for crops and plants is a relatively emerging technology. While it has gained substantial attention and momentum within the research community over recent years, its adoption at the end-user level, particularly among farmers, remains limited. This hesitation can be attributed to several factors, including insufficient technical expertise to operate and interpret drone-derived data, as well as the additional financial burden associated with acquiring and maintaining the required equipment. Drones (UAVs) are mainly chosen to monitor plot-level yield estimation. They can be equipped with sensors like multispectral, hyperspectral, LiDAR, TIR, and high resolution RGB. The use of these sensors enables researchers to collect high-resolution localized, spatiotemporal data, which can provide centimeter-level precision (Yuan et al., 2024; Gade et al., 2025). Such data typically encompass detailed information on crop and plant canopy attributes, as well as the physiological and structural properties of the underlying soil. The acquired datasets are subsequently employed by researchers to assess plant water balance and physiological responses to stress. In recent studies, there has been a growing tendency to rely predominantly on UAV-acquired data for training yield prediction models, based on the premise that manually collected datasets are more susceptible to human error, whereas UAV-derived measurements offer greater consistency and reliability. It can be concluded that, if fully adopted by farming communities, UAV-based methods can provide non-destructive and scalable forecasting of yield estimations for both crops and plants (Prey et al., 2022).

#### 2.2.3 Manned aerial systems

Manned Aerial Systems (MAS) consist of both the fixed-wing and the rotary-wing drones. These aerial systems are significant for agricultural surveillance because they can cover large areas. MAS operate at high altitudes, making it possible to effectively achieve moderate-resolution multispectral, hyperspectral, and thermal data. This moderate resolution helps in the accurate assessment of crop phenological dynamics at regional levels (Salomi et al., 2025). MAS also support extensive spatial coverage in data collection, which is vital for commercial agriculture. Their long-range endurance and scalability make them essential for predictive yield forecasting. This capability is realized through the systematic integration of acquired datasets into agro-informatics-driven decision-support systems for data-informed agronomic planning and management (Chen et al., 2023).

#### 2.2.4 Ground-based platforms

RS ground-based platforms have emerged as one of the pillars supporting agricultural yield estimating methods. They exist in various shapes and forms; for example, they can be mounted directly on harvesting equipment or designed and deployed as a crop-specific ground-based rovers equipped with advanced sensors. However, in some cases, researchers and users prefer fixed, static proximal sensors installed at strategically important locations within the field. The range of sensors supported by ground-based platforms is largely comparable to those integrated into UAV- and MAS-based systems. Their primary advantage, however, is that these sensors can have direct power supply and direct wired communication links instead of relying on WSNs (Gatkal et al., 2024). This configuration enhances reliability, data integrity, and transmission stability, making them a preferred option for data acquisition among both researchers and end users, including farmers. They can be relied upon in situations where WSN-based RS systems are limited. However, researchers and users have reported that ground-based platforms have low scalability compared to WSN-based RS systems. Despite these limitations, they enable high-resolution and, in many cases, near real-time assessment of phenotypic characteristics. The range of tasks they support includes, but is not limited to, analysis of soil moisture dynamics, monitoring of canopy temperature variations, and evaluation of localized microclimatic conditions. Some researchers are also validating the outputs of mobile dataset-based prediction models using these static datasets. This cross-validation approach enhances the calibration and robustness of radiative transfer models as well as ML-based predictive frameworks, ultimately improving their generalization and reliability. The integration of ground-based platforms can significantly boost the accuracy of yield prediction models for both crops and plants (Fei et al., 2023).

### 2.3 Analytical and modeling approaches

The analytical and modeling approaches opted by researchers for yield estimation use advanced statistical frameworks. Those frameworks rely on crop growth simulation models. ML algorithms can further support the prediction of productivity outcomes by using the collected multidimensional datasets. Such datasets include physiological traits, environmental drivers, and management variables. Together, these approaches enable robust and scalable yield forecasting in both the crops and plants.

#### 2.3.1 Vegetation indices

Most RS sensors deployed in agricultural settings are primarily designed to derive vegetation indices, commonly including the NDVI, the Soil-Adjusted Vegetation Index (SAVI), and the Green Normalized Difference Vegetation Index (GNDVI). These indices act as sophisticated spectral variables that can be used for yield estimation in both crops and plants (García Cárdenas et al., 2018; Basso et al., 2019). They accurately capture the variations in reflectance for both the visible and near-infrared range. These measurements can subsequently be utilized to derive key biochemical and biophysical attributes of the agricultural target under investigation. These characteristics include chlorophyll content, photosynthetic activity, and LAI. In this context, SAVI offers superior capability for soil background correction, while GNDVI provides enhanced sensitivity to chlorophyll absorption. Their reading-based datasets are considered essential components of modeling algorithms that support accurate yield estimation of both crops and plants (Awate and Nagne, 2023; Kaya and Polat, 2023).

#### 2.3.2 Crop growth simulation models

Crop growth simulation models are developed by researchers as advanced integrative frameworks that incorporate variables such as soil properties, genetic characteristics, and management practices to support accurate yield forecasting. Some of the widely used platforms include the Decision Support System for Agrotechnology Transfer (DSSAT), the Agricultural Production Systems Simulator (APSIM), and the World Food Studies (WOFOST) model. Researchers have pointed out that these simulation frameworks assimilate diverse datasets for yield estimations based on agricultural environments. Those diverse datasets may include soil-plant-atmosphere interactions, management strategies, and stochastic weather dynamics. It has been observed that the inclusion of stochastic weather dynamics-based assumptions and calculations increases the model’s reliability (Abayechaw, 2021; Gavasso‐Rita et al., 2024; George et al., 2024; Li et al., 2024). When these advanced simulation tools are supplied with RS-based vegetation index datasets derived from multispectral, hyperspectral, and thermal spectra, model performance is further enhanced. This integration ensures reliable calibration of canopy reflectance, LAI, and stress-related indicators. It also enables dynamic parameter assimilation and improved spatiotemporal scalability in both the localized and general level study on crops and plants. Ultimately, it has been observed that such integration establishes an adaptive, data-intensive, and analytically robust tool for yield forecasting in agricultural environments (Singh et al., 2025).

#### 2.3.3 Data fusion techniques

Data fusion techniques are another advancement for yield estimation in the agricultural sector. For example, integrating SAR and optical imagery facilitates data-fusion frameworks that capture structural and dielectric characteristics through SAR while simultaneously leveraging optical data to provide rich spectral information (Mustak et al., 2019). Researchers have emphasized hybrid and data-fusion strategies because such systems can effectively overcome the limitations inherent in individual sensors. This capability, in turn, ensures more consistent and uninterrupted data availability for end users, particularly farmers, even under adverse atmospheric conditions. Various approaches have been adopted by researchers for the fusion of data, including Bayesian approaches, radiative transfer models, and DL frameworks. The adoption of these approaches facilitates the precise extraction of crop biophysical and biochemical parameters, thereby enhancing prediction reliability and improving the scalability of yield forecasting systems (Sisheber et al., 2023).

#### 2.3.4 Phenology-based estimation

Phenology-based yield estimation operates as a process-driven analytical framework that employs multi-temporal RS observations to characterize crop developmental dynamics. These observations are linked to stage-specific phenological transitions that influence yield potential. Such relationships are examined through systematic analysis of growth stages, vegetative expansion, reproductive development, and senescence using spectral-temporal indices, canopy reflectance characteristics, and thermal signals (Pei et al., 2025). This approach supports accurate assessment of biomass distribution and stress-induced variability. Furthermore, incorporating time-series data into radiative transfer and ML models improves robustness by mitigating uncertainties associated with single-date observations, while the inclusion of phenological metrics enhances the predictive accuracy of crop and plant yield estimation. This temporally resolved methodology has the capacity to support high-resolution, scalable, and computationally robust yield forecasting systems, thereby reinforcing precision agro-informatics and sustainable agricultural intensification (Sakamoto et al., 2013).

**Figure 3:**
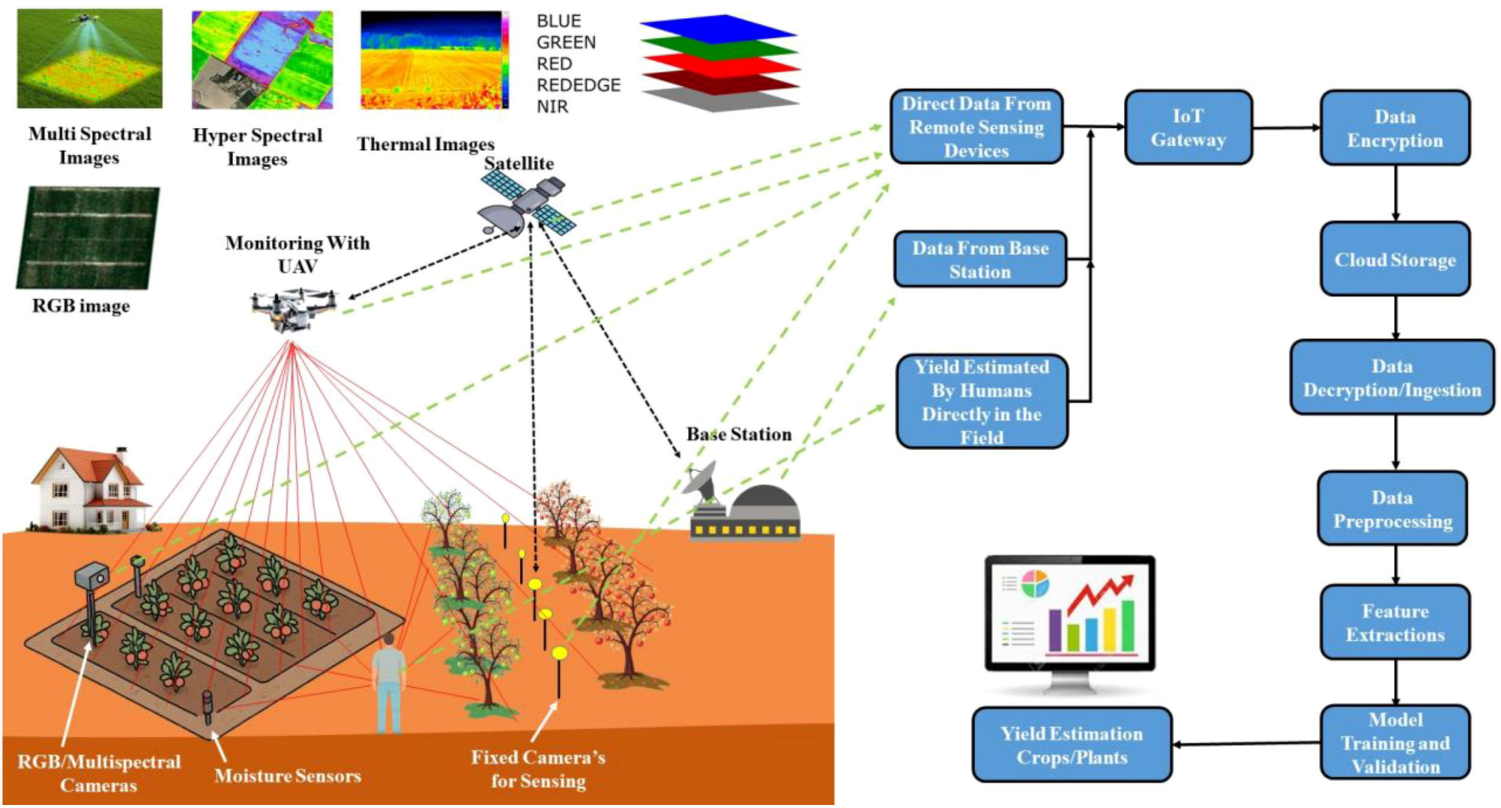
A basic scenario for yield estimation in an IoT-based framework applicable to both wireless and wired data collection.

To reinforce the preceding discussion, a schematic illustration and a basic layout have been included to aid the reader’s comprehension. **Figure 3** presents a simplified flow diagram that outlines the fundamental stages involved in information processing for yield forecasting.

### 2.4 Machine learning models

**Figure 4:**
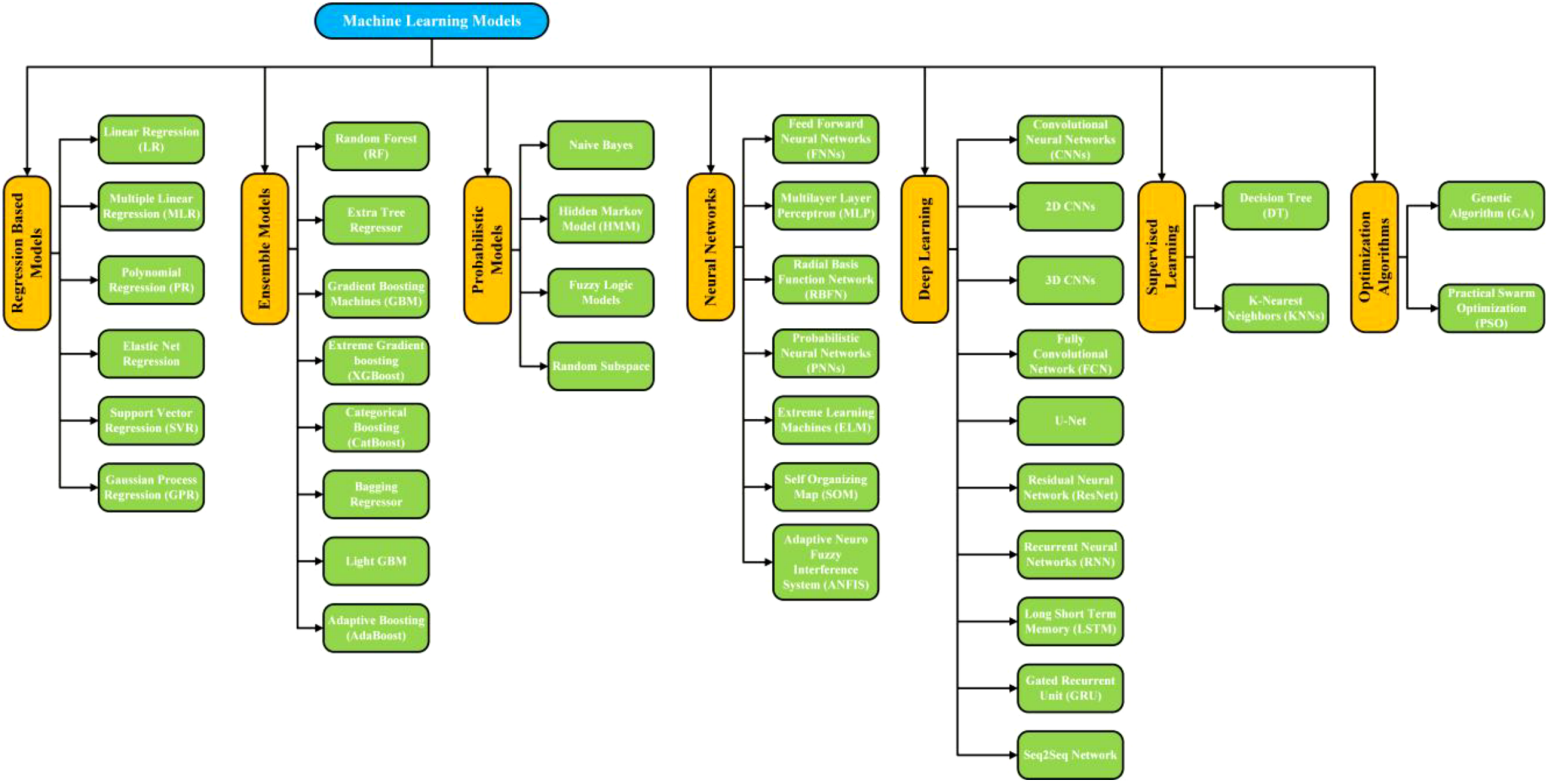
A flow diagram encapsulating the algorithms/methods used for the yield estimation in crops and plants.

This section is intended to critically synthesize how data-driven algorithms are employed to enhance the accuracy, robustness, and scalability of predictive tasks in RS-based agricultural applications. Rather than cataloging individual techniques, this section focuses on clarifying the functional role of ML in transforming high-dimensional, multi-source RS data into actionable agronomic insights. Emphasis is placed on explaining how different model families address non-linearity, uncertainty, and spatiotemporal variability, while also highlighting their assumptions, data dependencies, and practical limitations. Although ML approaches are part of the Analytical and Modeling Approaches discussed in Section 2.3, ML methodologies merit a dedicated section. Presenting them under a single short subheading would not adequately capture their complexity. To enhance clarity and reader comprehension, **Figure 4** provides a structured flow diagram that systematically illustrates the ML algorithms employed for crop and plant yield estimation. The major categories of ML techniques commonly employed include: (i) Regression-based models, (ii) Ensemble learning models, (iii) Probabilistic models, (iv) Neural networks, (v) Deep learning architectures, (vi) Supervised Learning Models, and (vii) Optimization Algorithms.

#### 2.4.1 Regression-based models

Regression-based techniques enable the formulation of explicit mathematical relationships between predictor variables. Among these, Linear Regression (LR) serves as the most straightforward and interpretable model. It can represent yield as a linear function of variables such as NDVI, canopy temperature, and soil moisture. Although LR is efficient in modeling simple linear dependencies, researchers have also reported that its performance deteriorates when yield responses exhibit non-linear behavior across the collected dataset (Iniyan et al., 2023). To address these challenges, Multiple Linear Regression (MLR) extends the LR framework by incorporating multiple independent variables. This extension enables the simultaneous evaluation of diverse spectral signatures and agronomic variables that collectively influence yield estimation (Piekutowska et al., 2021). MLR continues to be one of the most commonly used approaches in RS-based yield estimation, particularly under conditions where relationships are approximately linear but time predictor intercorrelations are minimal. However, agricultural datasets often demonstrate strong multicollinearity that in turns leads to the adoption of Polynomial Regression (PR), which integrates higher-order polynomial terms to model complex non-linear growth trajectories and phenological variations in crops (Jha et al., 2022). PR is particularly effective in capturing quadratic or exponential relationships, such as yield saturation at elevated vegetation index values. Nevertheless, its increased flexibility also leads to overfitting, especially when the dataset size is limited.

To overcome this issue of overfitting and multicollinearity, Lasso Regression (L1 regularization) is used to incorporate a penalty term into the loss function. This penalization suppresses less informative coefficients, which enhances the model’s stability, robustness, and overall reliability, especially under challenging conditions where strongly correlated predictors are prevalent, such as in multispectral or hyperspectral datasets (Ahmed et al., 2022; Gulshan et al., 2024). However, in contrast, the Ridge Regression (L2 regularization) promotes sparsity by reducing insignificant coefficients to zero, facilitating automatic feature selection, and improving model interpretability (Shafiee et al., 2021; Iniyan et al., 2023). On the other hand, the Elastic Net Regression, which merges both the L1 and L2 penalties, has the capability to provide a balanced mechanism between coefficient shrinkage and variable selection. This quality makes it highly suitable for yield estimation scenarios with numerous interrelated variables, such as vegetation indices that are derived from multiple spectral bands (Setiya et al., 2024; Rajendiran and Rethnaraj, 2025). Collectively, these regularized regression approaches are crucial for managing high-dimensional agricultural datasets, where redundant or noisy inputs may otherwise compromise the accuracy of traditional regression models.

Support Vector Regression (SVR) applies powerful kernel functions such as the radial basis function (RBF), polynomial, and sigmoid kernels. These methods project input data into higher-dimensional feature spaces, enabling the model to capture complex non-linear interactions between environmental variables and crop yield, which can then be represented and analyzed as linear relationships. This method offers strong resistance to outliers and superior generalization ability, ensuring accurate yield predictions across different spatial and temporal contexts (Kok et al., 2021).

Conversely, Gaussian Process Regression (GPR) adopts a probabilistic modeling perspective, treating yield as a probability distribution rather than a fixed outcome, thereby inherently capturing predictions uncertainties in yield estimation (Ghosh et al., 2022b; Lagrazon and Tan, 2023). Together, regression-based techniques, ranging from basic linear formulations to probabilistic models, have the capacity to form a comprehensive analytical toolkit for modern yield estimation. Such integration facilitates a balanced trade-off between model interpretability, adaptability to varying agronomic conditions, and predictive precision across diverse crop types. In **Table 2** we have provided a comparative performance analysis of the aforementioned regression-based methods.

**Table 2:** Comparative analysis of regression-based models used for yield estimation in crops and plants.

S.No	Metric	(LR)	(MLR)	(PR)	(L2)	(L1)	(L1+L2)	(SVR)	(GPR)
1	Accuracy (%)	75–82	78–85	80–88	83–90	84–91	85–92	88–94	89–95
2	Precision (%)	74–81	77–84	79–87	82–89	83–90	84–91	87–93	88–94
3	Recall (%)	72–80	75–83	78–86	82–88	83–90	84–91	86–93	88–95
4	F1-Score (%)	73–81	76–84	79–87	82–89	83–91	84–92	87–94	88–95
5	Training Time	Very Fast	Fast	Medium	Fast	Fast	Medium	Medium	Slow
6	Inference Time	Very Fast	Fast	Medium	Fast	Fast	Medium	Medium	Slow
7	Model Complexity	Very Low	Low	Medium	Medium	Medium	Medium	High	High
8	Robustness to Noise	Low	Low–Medium	Medium	High	High	High	High	Very High
9	Scalability	Very High	High	Medium	High	High	High	Medium	Low
10	Interpretability	High	High	Medium	High	High	High	Low	Low

#### 2.4.2 Ensemble learning models

Ensemble learning models have emerged as a crucial component of modern agricultural data analysis, especially for crop and plant yield prediction. This is due to their strong capability to combine multiple weak or base learners into a unified and highly effective predictive framework. This methodology effectively minimizes overfitting, strengthens generalization, and improves prediction reliability when processing diverse, complex, and noisy datasets originating from RS.

Random Forest (RF) integrates multiple decision trees through the bagging (bootstrap aggregation) approach. In that approach, each tree is trained independently on randomly chosen subsets of data and features. The ensemble’s overall prediction is then derived by averaging results for regression problems or by majority voting in classification tasks. In the context of yield estimation, RF exhibits outstanding robustness against noise and multicollinearity in the datasets. This robustness efficiently helps in modeling complex non-linear interactions among vegetation indices, soil moisture, temperature, and crop biomass (Prasad et al., 2021; Dhillon et al., 2023). The Extra Trees Regressor (ETR) advances this concept by introducing greater randomness in feature selection and threshold determination. This helps in enhancing computational efficiency and reducing bias toward dominant variables by making it particularly suitable for analyzing high-dimensional hyperspectral data (Sudhamathi and Perumal, 2024).

Boosting-based ensemble techniques, such as Gradient Boosting Machine (GBM), XGBoost, LightGBM, and CatBoost, construct additive models in a sequential manner, where each new learner attempts to correct the residual errors of the previous ensemble. GBM iteratively minimizes loss functions by using gradient descent, which helps provide exceptional flexibility and high predictive precision in crop yield estimation (Huber et al., 2022; Kulpanich et al., 2023; Uribeetxebarria et al., 2023; Wang et al., 2025). XGBoost extends the qualities of GBM through optimized regularization, parallelized computation, and advanced handling of sparse data. These enhancements collectively improve accuracy and scalability in a way that is particularly useful in large-scale UAV- or satellite-based yield monitoring (Kulpanich et al., 2023). LightGBM, designed for speed and efficiency, employs leaf-wise tree growth and histogram-based splitting, achieving superior performance with lower memory usage, which is an advantage in edge computing applications for smart farming (Wang et al., 2025). On the other hand, CatBoost is developed to efficiently handle categorical variables. It mitigates target leakage through ordered boosting and permutation-based encoding, making it valuable for mixed agronomic datasets that contain both continuous and categorical inputs in cro and plant yield estimation (Uribeetxebarria et al., 2023). Additionally, Adaptive Boosting (AdaBoost) adjusts the weight of each training instance based on previous errors, giving greater importance to unpredicted samples in subsequent iterations. This dynamic weighting mechanism allows AdaBoost to excel in scenarios with imbalanced agricultural data or rare crop stress occurrences (Faizan and Khilar, 2025). However the Bagging Regressor can enhance robustness by training several base regressors on random subsets and averaging their outputs, effectively reducing variance and improving stability in yield prediction in both crops and plants (Yadav et al., 2024; Yan et al., 2025). **Table 3** provides a comparative performance analysis of the Ensemble Learning- and Supervised learning Models-based methods.

**Table 3:** Comparative analysis of ensemble and supervised learning models used for yield estimation in crops and plants.

S.No	Metric	(DT)	(RF)	(ETR)	(GBM)	XGBoost	LightGBM	CatBoost	AdaBoost	Bagging regressor
1	Accuracy (%)	75–82	88–94	87–93	89–95	90–96	91–96	90–95	86–92	85–91
2	Precision (%)	74–81	87–93	86–92	88–94	89–95	90–95	89–94	85–91	84–90
3	Recall (%)	73–80	86–92	85–91	87–93	88–95	89–95	88–94	84–90	83–89
4	F1-Score (%)	73–81	87–93	86–92	88–94	89–95	90–95	89–94	85–91	84–90
5	Training Time	Very Fast	Medium	Fast	Medium-High	High	Very Low	Medium	Medium	Low–Medium
6	Inference Time	Fast	Fast	Fast	Medium	Medium	Very Fast	Fast	Fast	Fast
7	Model Complexity	Low	Medium	Medium	High	High	High	Medium	Medium	Medium
8	Robustness to Noise	Medium	High	High	High	Very High	Very High	Very High	Medium–High	High
9	Scalability	Medium	High	High	High	Very High	Very High	High	Medium	High
10	Interpretability	High	Medium	Medium	Low-Medium	Medium	Medium	High	Medium	Medium

#### 2.4.3 Probabilistic models

Probabilistic modeling approaches are also a powerful analytical tool used in agricultural yield estimation due to their capacity to incorporate uncertainty, variability, and non-linearity inheritance in agroecosystems. Unlike most of the deterministic models, which assume fixed relationships between inputs and outputs, probabilistic models treat crop yield prediction as a stochastic process influenced by numerous interacting factors. These factors may include soil moisture, spectral indices, temperature fluctuations, and phenological variations.

Naive Bayes (NB), based on Bayes’ theorem, models the conditional probability of yield outcomes given specific input features under an independence assumption. Despite its simplicity, NB performs remarkably well in early-stage yield classification, disease detection, and stress condition assessments where probabilistic relationships can be approximated efficiently (Chandana and Parthasarathy, 2022).

Hidden Markov Models (HMMs) extend probabilistic reasoning into the temporal domain by capturing sequential dependencies among growth stages and environmental states. They model crop yield evolution as a sequence of states, such as germination, vegetative growth, flowering, and maturation, where each is associated with observable variables such as LAI, NDVI, or canopy temperature. This temporal modeling capability makes HMMs particularly useful for monitoring yield dynamics across the growing season, using specifically time-series satellite data (Taylor and Browning, 2022). Similarly, Fuzzy Logic Models also have the capability to emulate human reasoning to handle ambiguity and imprecision in agricultural data. By defining fuzzy membership functions for variables such as high NDVI or moderate soil moisture, Fuzzy inference systems enable interpretable decision-making frameworks that capture gradual transitions in plant health. This approach allows reliable yield estimation under uncertain or incomplete sensor information (Kuzman et al., 2021).

Hybrid probabilistic ensemble methods, such as the Random Subspace Method (RSM), combine probabilistic sampling with ensemble learning to improve model robustness and generalization. The RSM constructs multiple base classifiers by training them on randomly selected subsets of features, thereby reducing variance and mitigating overfitting in high-dimensional datasets (Hazra et al., 2023; Ghochanian Haghverdi et al., 2024). By integrating these methods with multisource data from sources such as UAV imagery, soil sensors, and meteorological records, researchers can develop more reliable, interpretable, and adaptive yield estimation models that advance precision agriculture and sustainable farming practices. **Table 4** provides a comparative performance analysis of the Probabilistic, Supervised Learning Models, and Optimization Algorithms-based methods.

**Table 4:** Comparative analysis of probabilistic, supervised learning models and optimization algorithms used for yield estimation in crops and plants.

S.No	Metric	(k-NNs)	(NB)	(HMM)	(FLM)	(GA)	(PSO)	(RSM)
1	Accuracy (%)	85–90	80–86	86–92	84–90	88–93	89–94	90–95
2	Precision (%)	83–88	78–85	84–90	82–88	86–92	87–93	88–94
3	Recall (%)	84–89	79–86	85–91	83–89	85–91	86–93	89–95
4	F1-Score (%)	84–89	79–85	85–91	83–89	86–92	87–93	89–94
5	Training Time	Medium	Very Fast	Medium	Medium	Slow	Medium	Fast
6	Inference Time	Slow	Very Fast	Medium	Fast	Medium	Fast	Fast
7	Model Complexity	Moderate	Low	High	Moderate	High	Moderate–High	High
8	Robustness to Noise	High	Moderate	High	High	Very High	Very High	Very High
9	Scalability	Low	High	Medium	Medium	High	Very High	Very High
10	Interpretability	Medium	High	Medium	Very High	Medium	Medium	Low

#### 2.4.4 Neural network

Neural network (NNs)-based models have emerged as powerful computational tools for yield estimation due to their ability to approximate complex, non-linear, and high-dimensional relationships among environmental, spectral, and agronomic variables. Unlike conventional statistical models that rely on explicit functional assumptions, NNs have the capability to learn from data through iterative optimization, capturing intricate dependencies among soil moisture, vegetation indices, temperature, rainfall, and canopy reflectance. Among the most widely used architectures, the Feed-Forward Neural Networks (FNNs) serve as foundational models. They consist of an input layer, one or more hidden layers, and an output layer. Each neuron in FNNs applies an activation function to the weighted sum of the inputs, enabling non-linear transformations that map raw sensor or spectral data to yield predictions. Their simplicity and adaptability make them ideal for continuous regression tasks, such as biomass and grain yield estimation across varying phenological stages (Chang et al., 2024). Building upon the FNNs, the Multilayer Perceptron (MLP) introduces multiple hidden layers and non-linear activation functions. This structural enhancement significantly augments its representational capacity, enabling the modeling of complex agro-ecological interactions and non-linear system dynamics. MLPs are frequently employed in RS-based yield estimation modeling where-multi source inputs, such as NDVI, canopy temperature, and soil nitrogen content, require hierarchical feature extraction. Training is generally carried out using the backpropagation algorithm, which iteratively reduces the mean squared error between the predicted yields and the observed ground-truth values (Tripathi et al., 2022).

In contrast, to the MLP, the Radial Basis Function Network (RBFN) adopts a different activation strategy by employing Gaussian or radial functions that measure the distance between input vectors and prototype centers. This structure allows RBFNs to achieve faster training convergence and superior performance on localized or non-linear yield patterns, making them suitable for site-specific precision agriculture applications (Wu et al., 2022).

Probabilistic Neural Networks (PNNs) extend traditional neural models into a probabilistic domain by using kernel-based estimations of probability density functions. PNNs are particularly effective for classification that is oriented towards accurate yield estimation tasks. Those estimation tasks include distinguishing between high- and low-yield zones. This is accomplished by estimating the likelihood of each class and selecting the most probable outcome (Akanksha et al., 2021).

Similarly, the Extreme Learning Machine (ELM) offers an efficient alternative to gradient-based training by randomly assigning hidden-layer parameters and analytically determining output weights. This adaptation drastically reduces training time while maintaining high generalization accuracy, enabling rapid yield prediction from large-scale RS-based datasets (Vashisht et al., 2023).

However, the Self Organizing Map (SOM) approach has the capability to provide powerful visualization and clustering capabilities by mapping multidimensional input features onto a low-dimensional grid. In yield estimation, SOMs can identify spatial and temporal yield patterns, classify crop health zones, and detect anomalies in spectral signatures caused by stress factors such as drought or nutrient deficiency (Bustos-Korts et al., 2022).

Finally, the Adaptive Neuro-Fuzzy Inference System (ANFIS) integrates the learning capabilities of NNs with the interpretability of fuzzy logic. By representing knowledge in the form of fuzzy rules and membership functions, ANFIS can handle uncertainty and imprecision in agricultural data, producing explainable and robust yield predictions (Borse et al., 2025).

**Table 5** provides a comparative performance analysis of the NN-based methods discussed above.

**Table 5:** Comparative analysis of neural network-based models used for yield estimation in crops and plants.

S.No	Metric	(FFNN)	(MLP)	(RBFN)	(PNN)	(ELM)	(SOM)	(ANFIS)
1	Accuracy (%)	85–90	88–94	86–91	83–89	87–92	80–86	89–95
2	Precision (%)	83–89	87–93	84–90	82–88	86–91	79–85	88–94
3	Recall (%)	82–88	86–92	83–89	81–87	85–90	78–84	87–94
4	F1-Score (%)	84–89	87–93	84–90	82–88	86–91	79–85	88–94
5	Training Time	Medium	Slow	Fast	Medium	Very Fast	Medium	Slow
6	Inference Time	Fast	Medium	Fast	Fast	Very Fast	Medium	Medium
7	Model Complexity	Medium	High	Medium	Medium	Low	Medium	High
8	Robustness to Noise	Moderate	High	High	High	Moderate	High	Very High
9	Scalability	High	High	Medium	Medium	Very High	Medium	Medium
10	Interpretability	Medium	Low	Medium	High	Medium	High	Very High

#### 2.4.5 Deep learning architectures

Deep learning (DL) architectures have the capacity to automatically extract hierarchical and discriminative features from high-dimensional and heterogeneous agricultural data. Among these, Convolutional Neural Networks (CNNs) are widely adopted due to their exceptional performance in processing spatial and spectral data. Such data is commonly acquired from UAV platforms, satellite imagery, and ground-based sensing systems. CNNs effectively capture spatial patterns in canopy structure, vegetation indices, and texture features, enabling accurate mapping between visual cues and crop yield (Yang et al., 2021). Two-Dimensional CNNs (2D-CNNs) primarily handle spatial imagery such as RGB, multispectral, and hyperspectral data. However, when spectral and temporal information is integrated, Three-Dimensional CNNs (3D-CNNs) outperform their 2D counterparts by applying 3D kernels across spatial-spectral-temporal domains. The application of specific kernels allows for better representation of plant growth dynamics, phenological transitions, and stress responses over time (Nejad et al., 2022; Morales et al., 2023).

However, for pixel-level yield prediction and segmentation tasks, Fully Convolutional Networks (FCNs) and U-Net architectures are particularly valuable. FCNs replace traditional dense layers with convolutional layers to preserve spatial resolution, enabling the generation of dense yield maps that provide continuous spatial yield estimations. U-Net, with its encoder and decoder structure, has the capability to enhance localization accuracy by merging low-level spatial details with high-level semantic information. This fusion mechanism makes it particularly well-suited for plant canopy segmentation and fine-grained yield mapping, especially in spatially heterogeneous agricultural environments (Alkhudaydi and De la Lglesia, 2022; Buttar, 2024; Lei et al., 2024).

Residual Networks (ResNets) further advance the process of yield estimation by mitigating the vanishing gradient problem through shortcut (residual) connections. This design enables the effective training of very deep network architectures, thereby enhancing generalization capability and feature abstraction in complex agricultural settings. Such environments are characterized by high data variability resulting from soil background effects and crop heterogeneity (Fathi et al., 2023).

However, beyond spatial modeling, Recurrent Neural Networks (RNNs) are employed to model temporal dependencies within time-series datasets, including phenological growth patterns, weather sequences, and multi-temporal RS observations (Ingole et al., 2024). Traditional RNNs often suffer from long-term dependency issues, which are effectively addressed by Long Short-Term Memory (LSTM) networks. LSTMs incorporate gating mechanisms, input, and output gates, to regulate information flow, thereby retaining long-range temporal correlations that may be critical for predicting yield trends over entire growth seasons (Mateo-Sanchis et al., 2023).

Similarly, Gated Recurrent Units (GRUs) provide a simplified yet efficient variant of LSTM, achieving comparable accuracy with fewer parameters. This quality makes GRUs computationally suitable for large-scale agricultural datasets or edge-deployed smart farming systems (Alibabaei et al., 2021; Hussein et al., 2025).

However, in order to capture sequential input-output relationships more effectively, Sequence-to-Sequence (Seq2Seq) networks are leveraged for yield forecasting tasks, such as when there is use of multi-step yield prediction from sequential satellite images or weather data. In this framework, the encoder processes input sequences, such as spectral temporal features, while the decoder predicts future yield or growth states. This encoder-decoder paradigm effectively captures and models complex temporal dependencies and dynamic transformations within agricultural time-series data. This aforementioned architecture can be integrated with attention mechanisms, enabling the model to focus on critical phenological periods that influence yield outcomes (Orduna-Cabrera et al., 2025). **Table 6** provides a comparative performance analysis of the DL architectures discussed above.

**Table 6:** Comparative analysis of deep learning architectures based models used for yield estimation in crops and plants.

S.No	Metric	CNN	2D-CNN	3D-CNN	FCN	U-Net	ResNet	RNN	LSTM	GRU	Seq2Seq
1	Accuracy (%)	88–93	89–94	91–96	90–95	92–97	93–98	86–91	90–95	89–94	91–96
2	Precision (%)	87–92	88–93	90–95	89–94	91–96	92–97	85–90	89–94	88–93	90–95
3	Recall (%)	86–91	87–92	89–95	88–94	90–96	91–97	84–89	88–94	87–92	89–95
4	F1-Score (%)	87–92	88–93	90–95	89–94	91–96	92–97	85–90	89–94	88–93	90–95
5	Training Time	Medium	Medium	High	High	High	High	Medium	High	Medium	Very High
6	Inference Time	Fast	Fast	Medium	Medium	Medium	Medium	Medium	Medium	Fast	Medium
7	Model Complexity	High	High	Very High	Very High	Very High	Very High	Medium	High	Medium	Very High
8	Robustness to Noise	High	High	Very High	High	Very High	Very High	Medium	Very High	High	Very High
9	Scalability	High	High	Medium	Medium	Medium	High	High	High	Very High	High
10	Interpretability	Low	Low	Low	Low	Low	Low	Medium	Medium	Medium	Low

#### 2.4.6 Supervised learning models

Within this context, the Decision Tree (DT) acts as a core foundational learning model, dividing the input space recursively according to feature-based thresholds. DTs provide clear interpretability and highlight hierarchical feature importance. However reliance on a single tree structure often results in high variance and a tendency toward overfitting, particularly in intricate agricultural datasets (Kalichkin et al., 2021; Lillo-Saavedra et al., 2022).

On the other hand, k-Nearest Neighbors (k-NNs) offers a non-parametric approach in which yield is inferred by averaging the outputs of the most similar data points within the feature space. Its simplicity and adaptability to non-linear relationships make k-NNs effective for localized yield mapping using multispectral and hyperspectral features (Dsouza and Babu, 2024).

#### 2.4.7 Optimization algorithms

Beyond statistical reasoning, optimization that may be based on probabilistic models, such as the Genetic Algorithm (GA) and Particle Swarm Optimization (PSO), have gained prominence in crop yield prediction modeling processes. GAs mimic the process of natural selection by evolving a population of candidate solutions toward optimal regression or classification models that minimize prediction errors. They are particularly advantageous for feature selection, hyper parameter tuning, and optimizing non-linear model parameters in high dimensional agricultural datasets (Bi and Hu, 2021). On the other hand, PSO, inspired by the social behavior of bird flocking, represents algorithms where particles iteratively update their positions based on personal and collective best experiences. In yield estimation, PSO has been employed to optimize NN weights, regression coefficients, and vegetation index thresholds. This practice results in enhanced convergence accuracy across diverse crops in agro-climatic zones (Gong et al., 2023; Reddy and Madapuri, 2024).

## 3 Limitations in remote sensing-based yield estimation

**Figure 5:**
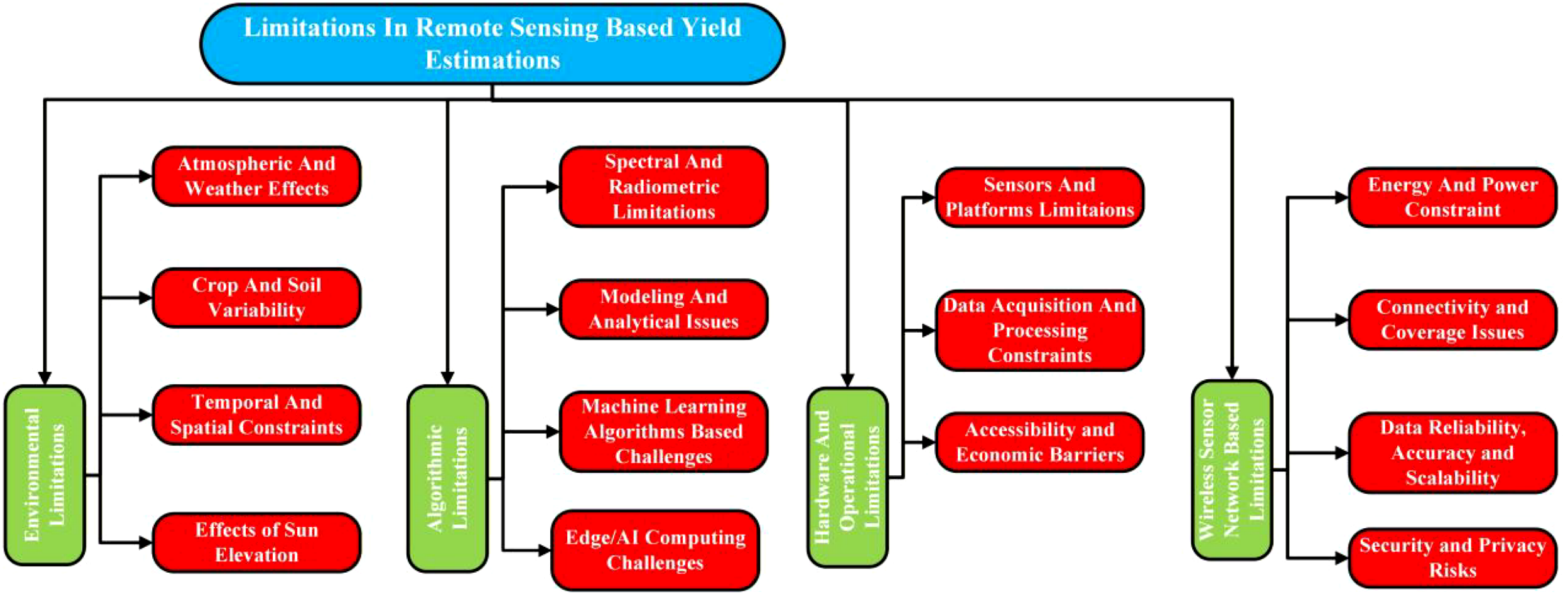
Categorization of the limitation in remote sensing-based yield estimation of crops and plants.

This section critically examines the limitations in RS-based yield estimation in order to provide a balanced and realistic understanding of current methodological constraints. Its primary purpose is to identify key challenges that hinder the accuracy, robustness, and scalability of yield prediction models. These limitations include sensor noise, atmospheric disturbances, spatial and temporal resolution trade-offs, data sparsity, and model generalization issues across crops and regions. By systematically outlining these constraints, this section establishes a clear context for interpreting reported results, highlights unresolved research gaps, and motivates the need for advanced sensing strategies, data-fusion frameworks, and more resilient modeling approaches. This section is divided into four categories: (i) Environmental Limitations, (ii) Algorithmic Limitations, (iii) Hardware and Operational Limitations, and (iv) Wireless Sensor Network-Based Limitations. **Figure 5** presents a flow diagram summarizing the key limitations in RS-based yield estimation.

### 3.1 Environmental limitations

Environmental challenges in RS-based yield estimation arise from factors such as atmospheric disturbances, seasonal variability, soil moisture fluctuations, and heterogeneous microclimates that degrade data quality and complicate spectral measurements.

#### 3.1.1 Atmospheric and weather effects

Regional weather fluctuation and rapidly changing atmospheric conditions have a major impact on RS-based yield estimations. These tend to introduce radiometric, geometric, and temporal distortions into the datasets. When such degraded datasets are subsequently used as inputs to prediction models, their estimation accuracy is significantly reduced. Among the atmospheric conditions, cloud cover has the most detrimental impact on the performance of optical sensors. Cloud presence significantly attenuates or completely blocks incident and reflected optical electromagnetic radiation, thereby limiting optical sensor effectiveness. In contrast, SAR systems operate in the microwave spectrum, allowing them to penetrate cloud cover and support all-weather data acquisition (Kordi and Yousefi, 2022; Cunha et al., 2024). Similarly, the occurrence of a localized dust storm can lead to the accumulation of dust particles on sensor surfaces, which directly degrades the accuracy of the acquired measurements. Such unavoidable weather-induced conditions introduce temporal data gaps and cause spectral distortions, ultimately compromising the continuity and reliability of the sensed data (Sillmann et al., 2021). Haze and atmospheric aerosols also cause scattering and absorption, which reduces the Signal-to-Noise Ratio (SNR). Changes in solar illumination geometry and topography-driven shadows generate anisotropic reflectance, undermining the radiometric comparability of multi-temporal datasets. All these factors substantially undermine the reliability of the sensor-acquired data, particularly when the measurements are influenced by the previously mentioned regional climatic or atmospheric variations. However, the research community has emphasized the use of advanced physics-based correction frameworks and the adoption of multi-angular sensing strategies. For dust and other organic particles, such as bird dropping, self-cleaning setups are recommended. Such measures facilitate the acquisition of high-fidelity sensor data, and the improved data integrity subsequently enhances the robustness and predictive reliability of yield estimation models for crops and other plant systems (Proctor, 2021).

#### 3.1.2 Crop and soil variability

Crop and soil variability have a powerful impact on estimation of yields in both crops and plants. This variability introduces substantial spatial and structural heterogeneity, which complicates the accurate retrieval of key biophysical parameters. As a result, the increased heterogeneity can degrade model calibration, limit generalizability, and, ultimately, reduce the reliability of predictive yield estimation models. To understand this effect, consider the example of the same crop cultivated simultaneously at two different locations. If a single prediction model is deployed to estimate yield at both sites without accounting for location-specific variations in soil properties and crop-soil interactions, the resulting predictions are likely to be biased or inconsistent due to unmodeled local heterogeneity (Shuai and Basso, 2022). In such scenarios, researchers tend to adopt higher-frequency monitoring of local soil heterogeneity across different crop growth stages. This approach enables the development of more robust, integrated mechanisms that effectively reduce uncertainties in vegetation-derived signals. Variations in soil albedo, moisture, and organic matter levels can alter the spectral characteristics of crops. As a result, vegetation indices such as NDVI and GNDVI will become less effective in detecting subtle physiological differences. Besides natural factors that can induce effects on the crop’s variability and heterogeneity of soil, There are also human-induced factors, such as change in irrigation patterns heavily changing evapotranspiration patterns. This has a direct effect on the thermal infrared responses and microwave backscatter dynamics of the crops and plants. Another human-based factor is fertilizer application, which modifies nitrogen uptake and chlorophyll content that directly affects photosynthetic efficiency, leading to detectable changes in spectral reflectance and biochemical indices. Planting density is another factor, as it triggers competition for soil nutrients between vegetation under observation. This competitive interaction significantly influences canopy architecture and alters intra-canopy light distribution dynamics, thereby affecting overall physiological performance and spectral response characteristics. Together, these mentioned variables generate pronounced spatial and temporal complexities that challenge the robustness of single-sensor approaches. To address these constraints, users should adopt multi-sensor data fusion strategies and apply soil-corrected spectral indices. Researchers further emphasize that these approaches should be complemented by site-specific calibration to ensure reliable performance. This way, the end user will be able to predict a correct yield in the presence of both crop and soil variability factors (Farooq et al., 2023; Raza et al., 2023).

#### 3.1.3 Temporal and spatial constraints

Temporal and spatial limitations also greatly affect the predictability of yield estimation algorithms, particularly in RS-based scenarios. Satellite revisit intervals are temporal processes that often span from a few hours to up to 16 days (Hadria et al., 2006), depending on the satellite constellation and the services purchased by the research team/observers or the end user (farmer). Consequently, in an ongoing project, such dependencies can lead to temporal gaps in the collected datasets. Moreover, the occurrence of adverse weather conditions, such as persistent cloud cover during satellite overpass, can further delay or even prevent data acquisition, resulting in missed observation windows over agricultural areas. This limitation restricts users of the technology from collecting various important information related to yield estimation in crops and plants. Such information may include rapid phenological transitions and transient stress events that significantly influence productivity (Qiao et al., 2021). Moreover, abrupt seasonal variability can further intensify these limitations, as seasonal changes directly influence the vegetation indices being monitored. Such indices include NDVI, GNDVI, and EVI across different crop growth stages. From a spatial perspective, these challenges are compounded by difficulties in capturing intra-field heterogeneity, particularly in fragmented fields managed by smallholder farmers. All these temporal and spatial limitations decrease the authenticity/robustness of the data. Consequently, predictive models trained on such uncertain or noise-contaminated data exhibit diminished generalization capability and reduced forecasting accuracy (Bishop et al., 2022). This degradation ultimately compromises future yield estimation performance for both crop and plant systems. To mitigate these limitations, the research community has increasingly shifted toward hybrid sensing paradigms, including integrated satellite-UAV platforms and satellite-SAR fusion frameworks, to enhance data continuity, spatial resolution, and overall model resilience.

#### 3.1.4 Effects of sun elevation

Collecting data from the same agricultural area using RS at different times of the day introduces inconsistencies. This is because the angles of sunlight change throughout the day, having a direct effect on how crops reflect incident radiation. These changing light angles can influence the dynamics of radiometric, geometric, and physiological cues. It has also been observed that at lower elevations, diminished photon flux reduces illumination intensity and lowers the SNR (Zhu et al., 2018). This reduction increases the variability in spectral observations. Additionally, low solar elevation angles during morning and evening hours tend to produce elongated shadows. These shadow effects can lead RS-based sensors to inaccurately estimate fractional vegetation cover and the associated biomass parameters. Researchers have reported that this phenomenon directly influences the anisotropic scattering behavior of incident radiation, leading to alterations in the BRDF. Such modifications can compromise the stability and robustness of derived vegetation indices. Background contrast between soil and vegetation is also affected at lower angles. This is because lower angles make it difficult to perceive things under observation. Solar elevation angles also affect thermal radiation and reflectance, which directly hinders the reception of correct stress signals. To understand this, think about the early morning. The measurements observed at that time often underestimate canopy stress due to delayed heating. On the other hand, when the sun is at its peak at midday, excessive radiative loading can sometimes cause a wrong stress signal. To mitigate these limitations, researchers adopt strategies that emphasize temporal harmonization and the use of standardized RS-based datasets, thereby ensuring more consistent and reliable yield estimation for crops and plants (Shafiee et al., 2023; Sivakumar, 2023; Li et al., 2025a).

### 3.2 Algorithmic limitations

Algorithmic challenges arise when users do not properly address dimensionality and heterogeneity in datasets. A prediction model created using data from one part of the world for a particular crop cannot be immediately applied to the same crop in another part of the world. Variability factors that may influence outcomes must be considered on algorithmic levels.

#### 3.2.1 Spectral and radiometric limitations

Spectral and radiometric constraints constitute significant barriers in RS-driven yield estimations, as they can limit both the accuracy and scalability of yield estimation predictive systems in crops and plants. One principal spectral constraint arises from the limited band coverage of conventional multispectral sensors, frequently lacking the spectral resolution necessary to detect subtle variations in crop canopy morphology and structural heterogeneity (Abdul-Jabbar et al., 2023). This reduces the sensitivity to efficiently detect physiological stress responses and phenological transitions, thereby restricting the adaptability of models across diverse agricultural settings. Another major challenge is the saturation of vegetation indices in dense biomass conditions. Researchers have reported that high LAI and canopy closure have the tendency to induce asymptotic reflectance in red and near-infrared wavelengths. This non-linear response leads to pronounced variations in canopy vigor and yield potential, thereby reducing the discriminative effectiveness of conventional spectral indices. Radiometric inconsistencies further exacerbate these challenges, compounding the uncertainty in yield-related assessments. Variations caused by sensor calibration drift, atmospheric disturbances, and illumination changes can create reflectance discrepancies across sensor platforms. To avoid this limitation, integration of various data collection platforms may be required. However, if such integration is not carefully managed, errors may propagate throughout the workflow of yield estimation instead of improving accuracy (Abdelbaki and Udelhoven, 2022; Luo et al., 2022).

#### 3.2.2 Modeling and analytical issues

Inaccurate modeling and analysis also represent major obstacles to the development of reliable and scalable yield estimation techniques. One key reason is the prevalent assumption among many researchers that yield estimation can be adequately addressed using linear regression-based models alone. However, closer examination indicates that this assumption is overly simplistic and does not fully capture the complexity of the problem. While vegetation indices may be considered as a linear regression process, environmental factors such as soil variability and heterogeneity are non-linear and sometimes very complex in nature. If these issues are not adequately addressed, the resulting analytical models become especially vulnerable to a range of well-known constraints. These include spectral index saturation effects, variability in canopy architecture, and noise introduced by atmospheric disturbances or soil background reflectance. Collectively, these factors undermine the robustness and generalizability of the models, ultimately reducing their reliability when applied across diverse agro-ecological environments (Filippi et al., 2025). The prediction models show poor transferability between crop types, which is another observation made by researchers and end users. In particular, changes in geographic locations have an impact on stages of crop growth. Domain-specific variations in canopy architecture, pigment dynamics, and management practices challenge the validity of calibration transfer assumptions. This lack of adaptability significantly constrains the applicability of such algorithms for large-scale or global yield forecasting. Adding to the complexity are integration difficulties in multi-source and multi-sensor data fusion, where variations in spatial resolution, spectral range, temporal revisit frequency, radiometric calibration, and sensor-specific BRDF effects complicate data assimilation processes. **Table 7** presents a set of algorithms that address multi-source and multi-sensor data fusion challenges in precision agriculture. Collectively, these analytical constraints highlight the pressing need for physically grounded, domain-adaptive, and uncertainty-aware modeling frameworks that enables users to obtain robust, reliable, and scalable crop yield predictions (Ma et al., 2021; Sadenova et al., 2021).

**Table 7:** Solutions for multi-source and multi-sensor data fusion-based challenges in precision agriculture.

S.No	Data fusion challenge	Data sources involved	Solutions	Limitations and open challenges	References
1	Heterogeneous data representation	UAV RGB, multispectral, hyperspectral, SAR	Vision Transformer with modality specific embeddings	High computational cost; patch-size sensitivity	(Lin et al., 2023; Mehdipour et al., 2025)
2	Cross modal correlation learning	Multispectral + LiDAR	Multimodal Transformer with cross attention	Overfitting risk; alignment complexity	(Lu et al., 2024; Jácome Galarza et al., 2025)
3	Temporal misalignment	UAV+ IoT sensors	Temporal Transformer	High memory usage; limited interpretability	(Cheng et al., 2024; Saki et al., 2025)
4	Missing sensor data	Multispectral, thermal, soil sensors	Masked multimodal Transformers	Performance drop under persistent sensor loss	(Shen et al., 2022b; Lu et al., 2024)
5	High dimensionality	Hyperspectral + RGB	Attention based feature selection Transformers	Limited physical interpretability	(Clement, 2025; Saki et al., 2025)
6	Domain shift	Satellite, UAV, weather data	Domain adaptive multimodal Transformers	Requires multiple domain data	(Tunio et al., 2024; Zhang et al., 2025)
7	Noisy measurements	SAR + optical RS	Transformer based denoising autoencoders	Risk of suppressing subtle signals	(Isinkaye et al., 2025; Zhao et al., 2025)
8	Limited labeled data	Multi sensor RS + field samples	Self-supervised multimodal Transformers	Pretraining bias risk	(Zhang et al., 2022; Cao et al., 2025)
9	Edge constraints	UAV+ IoT nodes	Lightweight Transformers, pruning, distillation	Accuracy and latency trade off	(Huang et al., 2024; Li et al., 2025b)
10	Explainability	Multisource RS + agronomic data	Explainable Transformers	Non causal attention interpretation	(Kamal et al., 2025; Rahman et al., 2025)

#### 3.2.3 Machine learning-based challenges

ML-based approaches for estimating crop and plant yields are restricted by methodological and operational limitations. These constraints reduce their predictive accuracy and limit their ability to remain aligned with the dynamic biological processes occurring within the observed agricultural environment. Keeping this in view, researchers do their best when acquiring datasets for specific crops or plants. However, some natural variables still remain missing from ground-truth datasets that are used for training and validation. This discrepancy limits the learning model’s stability, which restricts its ability to fully comprehend the prediction problem at hand. Researchers have found that full natural randomness cannot be captured within a dataset. As a result, human-collected datasets are often prone to overfitting. Consequently, when such trained models are applied to diverse agricultural systems across different regions of the world for the same crop, the resulting predictions are unlikely to remain accurate or fully reliable (Kamilaris et al., 2017; Rashid et al., 2021). Furthermore, stakeholders also show concerns regarding sharing datasets with experts for training and validating purposes. Weak coupling between RS-derived spectral metrics and process-based crop growth models further complicates things. The main reason is that data-driven methods excel at correlation detection but lack the mechanistic rigor to simulate biophysical processes. Collectively, these limitations highlight the need for hybrid data-efficient ML-biophysical integration frameworks. Such advancements are essential for developing more robust, generalizable, and scalable yield forecasting systems across diverse crop and plant species (Morales and Villalobos, 2023).

#### 3.2.4 Edge/AI computing challenges

Among recent algorithmic advancements in precision agriculture, Edge Computing and Edge AI have gained increasing prominence. This is particularly evident in WSN- and UAV-enabled precision agriculture systems. Traditional cloud-centric architectures rely on continuous data transmission from distributed sensors and aerial platforms to remote servers. This dependence introduces major challenges related to bandwidth saturation, latency, energy consumption, and network reliability. These issues are particularly severe in rural and infrastructure-limited agricultural environments. Edge computing addresses these limitations by enabling localized data processing directly on sensor nodes, gateway devices, or onboard UAV processors (Srivastav and Das, 2025). By deploying lightweight ML and DL models at the edge, raw multispectral, hyperspectral, and environmental data can be pre-processed, filtered, and analyzed locally. As a result, only high-level features or decision-relevant information are transmitted to the cloud, which significantly reduces communication overhead and improves real-time responsiveness for applications such as stress detection, irrigation scheduling, pest monitoring, and yield estimation (Natraj et al., 2025). However, the adoption of Edge AI introduces new hardware and algorithmic constraints, including limited computational resources, memory capacity, and energy availability at the edge devices (Islam and Ghose, 2025). These constraints require the adoption of model compression, quantization, federated learning, and energy-aware inference strategies to enable practical deployment. Consequently, integrating Edge Computing into WSN- and UAV-based agricultural systems represents a key design trade-off between distributed intelligence, system scalability, and operational sustainability (Shaikh et al., 2025).

### 3.3 Hardware and operational limitations

Hardware and operational challenges in yield estimation arise from various sensor and platform limitations. Hardware limitations may include thermal drift in electronics, short-lived moving components, low SNR, and calibration drift. These operational errors may originate from calibration errors, payload restrictions, and inconsistent sensor performance.

#### 3.3.1 Sensor and platform limitations

These limitations are critical in many ways as they can limit the accuracy and reliability of the system. For example, fine spatial resolution can only be obtained when RS-based equipment operates close to the observed field. This requirement inherently constrains the capacity of researchers and end users, including farmers, from monitoring extensive agricultural areas within limited time frames. Moreover, if large areas are to be monitored within a shorter time frame, observations must be conducted from greater distances, which inevitably leads to a reduction in spatial resolution (Khanal et al., 2020; Muhammad et al., 2025). UAVs, while capable of delivering centimeter-level details, are hindered by available energy constraints as battery technology is still not as developed to support long operational flight time. Additionally, payload capacity of a normal accessible UAV is limited. Consequently, researchers and end users are often unable to integrate multiple heterogeneous sensors simultaneously within a single deployment mission. They must take turns and replace sensors to collect each specific type of data, further draining battery capacity. These limitations are further aggravated by external factors, including weather-related disruptions and regulatory restrictions. Although alternative technologies, such as radar and satellite-based RS, can be employed, they are also not without limitations. These approaches are often associated with high acquisition and licensing costs, along with constrained temporal revisit frequencies. Therefore, there is an urgent need to develop cost-effective, easily manufacturable, and resilient hardware solutions specifically engineered for agricultural yield estimation applications. Failure to address these issues can hinder effective data acquisition, particularly in resource-constrained agricultural systems that require cost-effective and operationally scalable yield estimation solutions (Lee et al., 2010; Longchamps et al., 2022).

#### 3.3.2 Data acquisition and processing constraints

Data collection and subsequent data processing both have substantial constraints with regards to accurate yield estimation. Advanced RS data acquisition processes are inherently energy-intensive, and this burden is further amplified by computationally demanding pre-processing and post-processing stages. Together, these factors significantly increase overall system energy requirements. UAV technology is currently the most commonly used RS-based technology in agricultural monitoring. However, due to ongoing limitations in energy storage technologies, these systems struggle to sustain extended operational durations. This hinders their ability to acquire data. To address this limitation, researchers have recommended optimized flight path strategies to improve mission planning and help balance overall power consumption during data acquisition operations. In addition, sensors such as hyperspectral, multispectral, and LiDAR systems generate large volumes of data (Joshi et al., 2023). These datasets must either be stored and processed onboard the platform or transmitted to an external facility for subsequent storage and computational analysis. This creates a computational bottleneck, primarily because the limited payload capacity of UAV platforms restricts their ability to carry high-performance processing units capable of real-time data storage and analysis. As a result, systems often have to rely on sending data to a base station (BS) for further processing, which requires more energy. Downstream processing at the BS may include atmospheric and geometric correction, BRDF normalization, and noise filtering, all of which are critical for maintaining data integrity. However, these procedures introduce processing delays that may hinder timely decision-making. Researchers have also reported additional constraints when advanced ML algorithms and biophysical modeling frameworks are adopted, as these approaches introduce substantial computational overhead and markedly increase processing complexity. Collectively, these challenges highlight the need to develop integrated systems capable of supporting both efficient data acquisition and real-time processing for yield estimation across crops and plants (Rashid et al., 2021; Ma et al., 2022).

#### 3.3.3 Accessibility and economic barriers

In areas with limited resources, the high cost and limited availability of advanced agricultural equipment present major challenges. Researchers and users (farmers) may lack access to high-resolution imagery RS-based sensor systems. This constraint, in turn, restricts their ability to perform advanced data acquisition operations necessary for generating detailed datasets of locally cultivated vegetation. This major shortcoming weakens the robustness and generalizability of predictive models at local spatiotemporal levels. Users and researchers in such regions often depend on companies that control satellite and UAV-based imagery. This dependence increases overall costs and reduces flexibility in terms of vendor lock-ins. Furthermore, it also diminishes data sovereignty for stakeholders (Van Wart et al., 2013). This limitation can be exacerbated for users if they suddenly require scalability while monitoring large areas with multiple equipment based on RS WSNs because in such a scenario they will require more computational power, storage capacity, and sophisticated analytical framework resources. Addressing these scalability issues require both capital investments and technical skills, which may not be readily available. All these technological limitations and economic disparities mainly affect small landholders. To address this issue, open-access imagery repositories are a must and should be developed. Such initiatives would facilitate broader data availability and support the implementation of cost-effective sensing solutions tailored to local agricultural environments. This approach would also reduce reliance on commercially controlled data repositories that operate under monopolistic structures. Consequently, it would benefit both the research community and end users (farmers) by enabling more equitable and scalable yield estimation across diverse agricultural sectors for both crops and plants (Battilani, 2014).

### 3.4 Wireless sensor networks-based limitations

In the near future, it is obvious that the use of WSNs will be inevitable if farmers want to deploy IoT-based infrastructures in the agricultural field. However, this will also introduce a range of associated limitations and challenges.

#### 3.4.1 Energy and power constraints

Energy and power constraints are one of the greatest issues in the adoption of WSNs to measure crop and plant yield. Sensor nodes are usually battery-powered and most commonly have limited storage capacity, restricting system lifespan and equipment reliability for gathering data. Continuous execution of intensive sensing tasks, combined with real-time transmission of data, accelerates power consumption and may cause nodes to fail prematurely. When a node fails prematurely, it effectively compromises the integrity and reliability of the entire data collection process (Sakthipriya and Naresh, 2022). Researchers have observed that energy constraints may have limited impact when monitoring small areas. However, when monitoring large farmlands, frequent battery replacement is required to complete the task at hand. If multiple nodes are deployed, frequent battery servicing or replacement introduces considerable logistical complexity and increased operational costs. Such practices should be avoided, particularly when the monitored area is remote or geographically difficult to navigate. Therefore, researchers have emphasized the necessity of creating energy-efficient communication protocols, along with the creation of new lightweight materials that can store more energy within batteries. Several research groups have additionally explored the integration of renewable energy harvesting solutions within agricultural systems. This approach aims to support the development of energy-efficient sensing and monitoring infrastructures, thereby enhancing the sustainability and operational longevity of yield estimation frameworks (Hu and You, 2022; Aggarwal et al., 2024).

#### 3.4.2 Connectivity and coverage issues

Reliable connection and adequate coverage are still major challenges for WSN-based RS systems, especially in remote terrains where basic WSN communication infrastructure does not exist. These limitations significantly hinder the capabilities of researchers to successfully assess yield estimation practices. Researchers have recommended deploying a large number of sensor nodes to effectively capture the non-homogeneous characteristics of farmlands in such scenarios. However, high-density deployments substantially increase both initial installation costs and long-term operational and maintenance expenses. In non-homogeneous areas, WSN electromagnetic signals are likely to be distorted and experience attenuation in dense vegetation (Chen et al., 2016). Abrupt change of the weather can also contribute to poor connectivity and coverage issues. These issues increase packet loss and bit error rates of the communicated information between nodes. This degradation directly affects the reliability of predictive models, particularly in scenarios where long-distance communication becomes a necessary requirement for completing agronomic tasks. In such situations, additional WSN infrastructure, including gateways, repeaters, or hop-based relay systems, may be necessary. However, reliance on auxiliary components further heightens network latency, power consumption, and operational expenditure, thereby complicating scalable deployment of RS-based WSNs for efficient yield estimation in crops and plants (Majumdar et al., 2021; Mowla et al., 2023).

#### 3.4.3 Data reliability, accuracy and scalability

Data reliability, accuracy, and scalability, when compromised, have been identified as major constraints of sensor-based yield estimation. Sensors are affected by various factors. The most prominent of which are environmental factors, including changes in temperature, excessive humidity, dust, and rainfall. These factors often lead to calibration drift and the introduction of systematic biases within the acquired measurements, thereby degrading overall data fidelity. Such distortions compromise the integrity of spatiotemporal datasets associated with the monitored field, ultimately resulting in incomplete data representations with diminished predictive capability. Additionally, scalability challenges arise when a large number of sensor nodes are required to operate in a coordinated manner. Algorithms originally designed for small-scale networks often fail to perform efficiently under such conditions. As scalability issues emerge, the network rapidly loses its capacity to manage tasks effectively and deliver required outputs to end users in a timely manner (Ferencz et al., 2004). In some cases, the volume of data collected may exceed the processing capacity of available units, which will fail to process the data. This inability leads to latency, jitter, and packet loss. Electromagnetic interference generated by coexisting communication devices can further disrupt data transmission channels, compromising link reliability and reducing overall network throughput. All these limitations negatively affect the accuracy, homogeneity, and temporality of agronomic data. In such cases, robust calibration strategies and effective interference mitigation techniques become critical for enabling scalable and reliable yield forecasting (Grassini et al., 2015).

#### 3.4.4 Security and privacy risks

Security and privacy risks represent another significant threat to the reliability of yield estimation data, particularly when such information is transmitted wirelessly within WSNs. The openness of communication and access to resources makes WSNs prone to various cyber-attacks, such as eavesdropping, jamming, denial-of-services, spoofing, and false data injection. These attacks compromise the confidentiality, integrity, and availability of crucial agricultural information (Gupta et al., 2020). A single compromised node in WSNs can operate as a malicious insider, supplying falsified inputs that skew feature extraction processes and misdirect ML-based prediction models. When an RS node in an agricultural WSN is compromised, the impact becomes significantly intensified due to its limited processing power and constrained energy reserves. As a result, such nodes are often unable to implement robust encryption and authentication mechanisms, thereby increasing their vulnerability. Therefore, most communication nodes deployments of agricultural WSNs have weak security controls and are continuously exposed to attack from eavesdroppers. In light of these weaknesses, research communities and end users (farmers) recognize the urgent need for lightweight yet robust security systems to protect precision agriculture yield estimation systems (Majumdar et al., 2021; Manoj et al., 2023).

**Figure 6:**
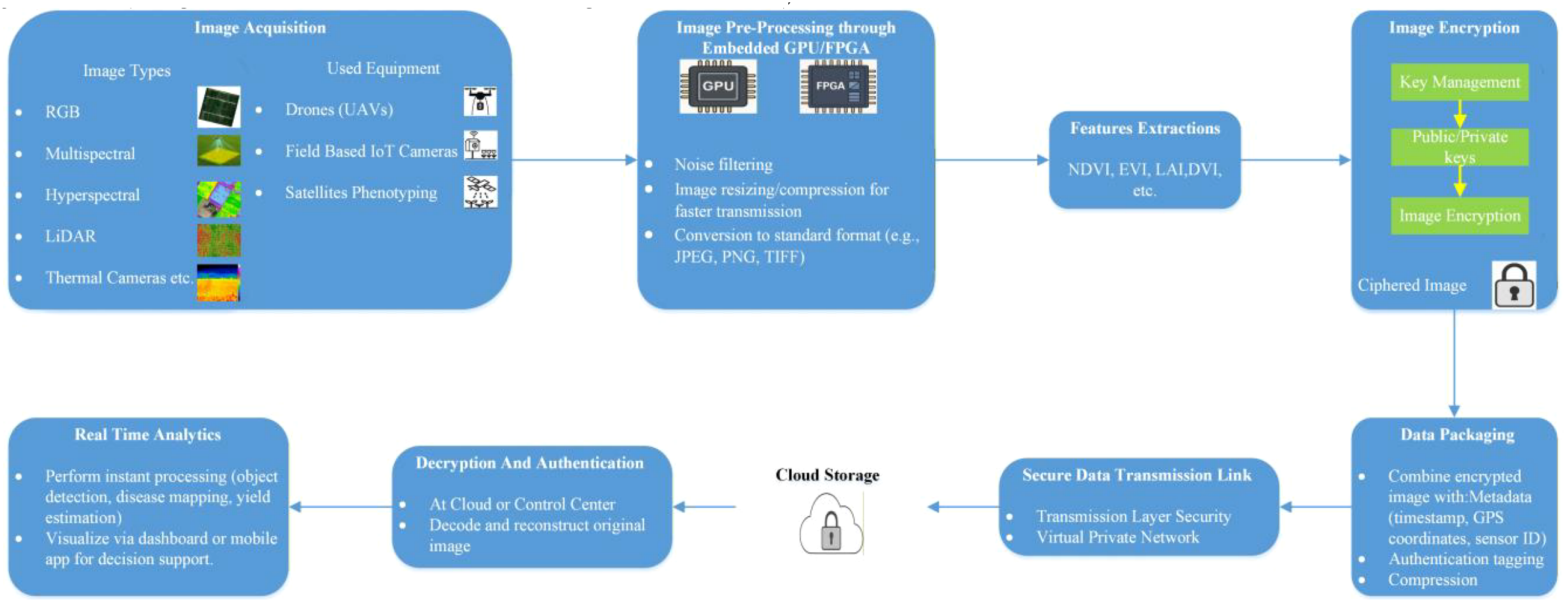
A basic flow diagram that depicts how cybersecurity works in real-time analysis for yield estimation in crops and plants.

In **Figure 6**, a generic flow diagram is presented to illustrate the key steps involved in achieving yield estimation, while simultaneously highlighting the measures required to ensure data security. The diagram demonstrates how data is protected as it traverses through the different stages of WSNs, from the transmitter (Tx) to the receiver (Rx). **Table 8** provides the categorical division of cybersecurity threats in yield estimation, along with example attacks and their impact on yield estimation.

**Table 8:** Cybersecurity attacks in smart farming and precision agriculture, types, impacts, and mitigation strategies.

S.No	Layer	Attack type	Description	Impact on agricultural systems	Mitigation strategies	References
1	Network Layer	Eavesdropping/Sniffing	Intercepting wireless communications (Wi-Fi, ZigBee, LoRa, BLE, etc.) to steal sensitive data.	Breach of soil, crop, or livestock data privacy.	Use strong encryption (AES, ECC), VPN tunneling, and secure communication protocols (TLS/DTLS).	(Barreto and Amaral, 2018; Gupta et al., 2020; Sontowski et al., 2020; Adewusi et al., 2022)
		Spoofing	Malicious node imitates legitimate sensor or gateway identity.	False data injection, automation errors.	Employ mutual authentication, digital certificates, and MAC based identity verification.	
		Replay Attack	Reusing captured valid data packets.	Misleading sensor readings or actuator responses.	Time stamping, nonce based authentication, and cryptographic MACs.	
2	Application Layer	Malware/Ransomware	Inserting malicious software to lock or hijack systems.	Inoperable smart devices, data loss.	Regular patching, antimalware tools, secure boot.	(Barreto and Amaral, 2018; Gupta et al., 2020; Sontowski et al., 2020; Van Der Linden et al., 2020)
		Phishing/Social Engineering	Deceptive emails or messages to steal credentials.	Unauthorized system access.	Employee awareness training by using MFA.	
		Data Injection/Manipulation	Altering data or ML model inputs.	Incorrect yield or irrigation predictions.	Data validation, checksum verification, model integrity audits.	
3	Physical Layer	Hardware Tampering	Physical modification of devices.	Sensor malfunction or sabotage.	Tamper proof enclosures, access control, surveillance.	(Barreto and Amaral, 2018; Gupta et al., 2020; Sontowski et al., 2020; El-Ghamry et al., 2023)
		Side-Channel Attack	Extracting cryptographic keys via power or EM analysis.	Compromise of secure keys.	Hardware security modules and resistant design.	
		Node Capture Attack	Capturing or reprogramming IoT devices.	Key theft, rogue nodes.	Secure boot loaders, encrypted storage, node attestation.	
4	Cloud & Data Layer	Data Breach	Unauthorized access to cloud data.	Exposure of yield, financial, or environmental info.	End-to-end encryption, access control, audits.	(Barreto and Amaral, 2018; Demestichas et al., 2020; Gupta et al., 2020; Sontowski et al., 2020)
		Model Poisoning	Corrupting ML datasets or parameters.	Degraded predictive accuracy.	Dataset integrity verification, differential privacy.	
		SQL Injection	Inserting malicious queries into inputs.	Database corruption or theft.	Parameterized queries, input validation, WAF.	
		Data Integrity Attack	Altering or deleting stored records.	Inaccurate farm history and analytics.	Blockchain logging, backups, checksum verification.	
5	Physical/Control Layer	Command Injection	Injecting false control commands to actuators.	Crop damage via over irrigation/pesticide misuse.	Command whitelisting, digital signatures, secure PLC firmware.	(Barreto and Amaral, 2018; Gupta et al., 2020; Sontowski et al., 2020; Zangana et al., 2025)
		Man-in-the-Middle (MITM)	Intercepting and altering transit data.	Manipulated UAV or sensor operations.	Strong authentication and TLS encryption.	
		Firmware Hijacking	Installing malicious firmware on devices.	Permanent system compromises.	Secure firmware updates and OTA authentication.	
6	AI/ML Layer	Adversarial ML Attack	Perturbing inputs to mislead ML models.	Wrong disease/stress detection.	Adversarial training, input sanitization, and robustness testing.	(Barreto and Amaral, 2018; Gupta et al., 2020; Sontowski et al., 2020; SP and Balamurugan, 2022)
		Data Poisoning Attack	Corrupting datasets during ML training.	Unreliable AI based predictions.	Secure data provenance, integrity checks, and anomaly filtering.	
		Model Extraction/Inversion	Reconstructing training data from outputs.	Leakage of proprietary datasets.	Differential privacy, API encryption, output limitation.	
7	Insider & Supply-Chain Layer	Insider Threat	Malicious actions by legitimate users.	Data theft, sabotage.	Access control, user monitoring, and insider threat detection.	(Barreto and Amaral, 2018; Linsner et al., 2019; Gupta et al., 2020; Sontowski et al., 2020)
		Supply Chain Attack	Exploiting vulnerabilities in vendor firmware or updates.	Hidden malware or backdoors in IoT devices.	Vendor auditing, firmware signing, third party risk assessment.	

## 4 Results analysis and discussion

**Figure 7:**
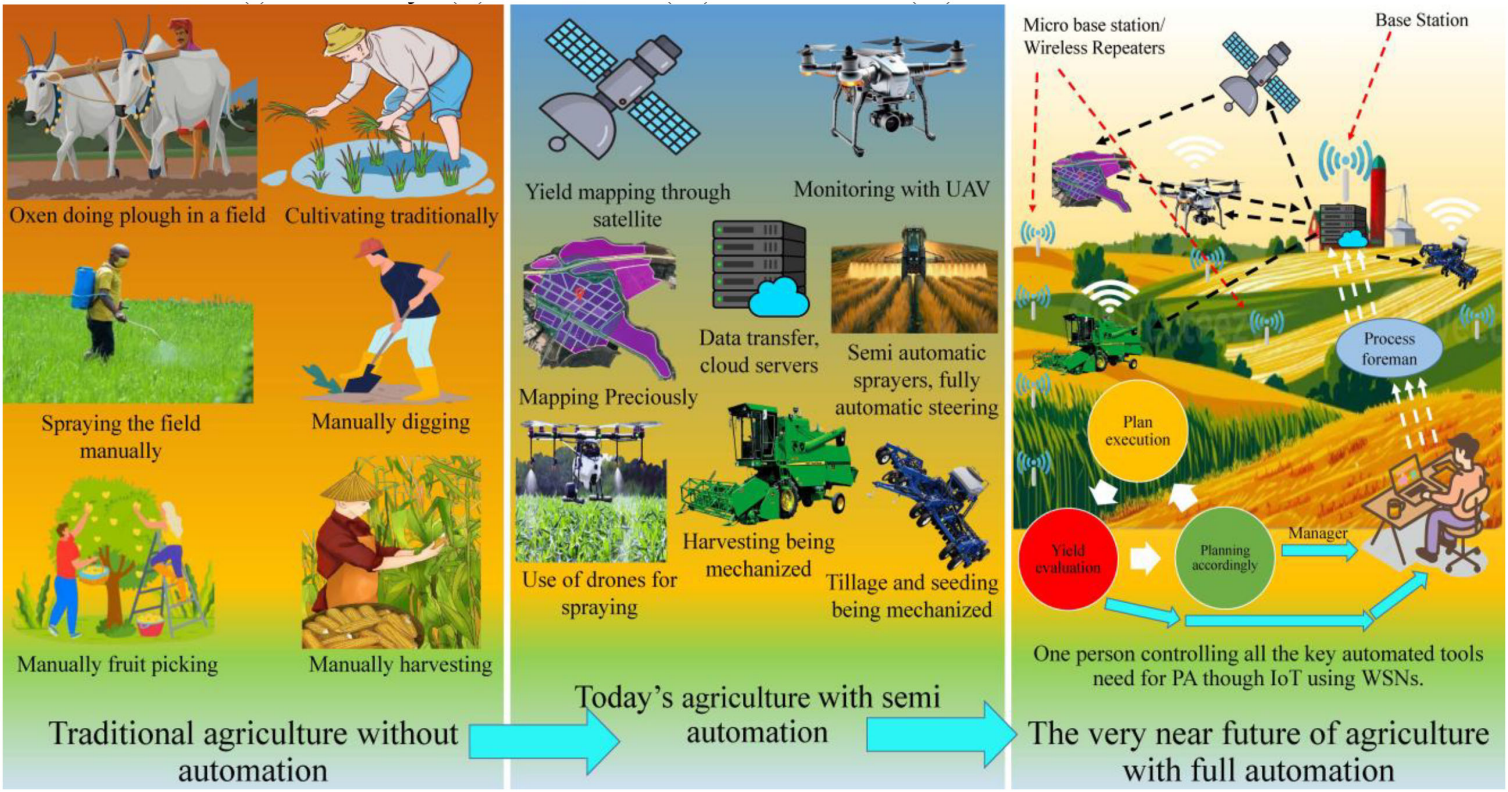
Advancements in agricultural practices with respect to time that ultimately help in yield estimation in crops and plants.

**Figure 7** illustrates the evolution of human agricultural practices over time. It also highlights the anticipated future of farming systems. This future may be driven by the integration of advanced technologies such as RS, WSNs, drones, satellites, and fully automated agricultural machinery connected through the IoT. The data generated from agricultural practices across different historical eras, as illustrated in the figure, are now being systematically organized and curated by various research groups. These carefully developed datasets are subsequently utilized by other researchers, who apply ML algorithms to achieve more accurate and reliable crop yield estimation. The Results Analysis and Discussion section is dedicated to critically interpreting the reported outcomes of ML-based studies in crop yield estimation in the form of bar graphs. The primary purpose of this section is not merely to summarize numerical performance metrics, but to provide a comparative and analytical understanding of how different ML models behave under varying data sources, crop types, and experimental conditions. By systematically examining indicators such as prediction accuracy, robustness, generalization capability, and computational efficiency, this section highlights the strengths, limitations, and practical implications of each modeling approach. This discussion enables readers to identify performance trends, methodological gaps, and informed directions for future research and real-world deployment. All the results presented in this section are related to the discussion provided in Section 2.4. The results are collectively compared using four key metrics: (i) Accuracy, (ii) Precision, (iii) Recall, and (iv) F1 score.

**Figure 8:**
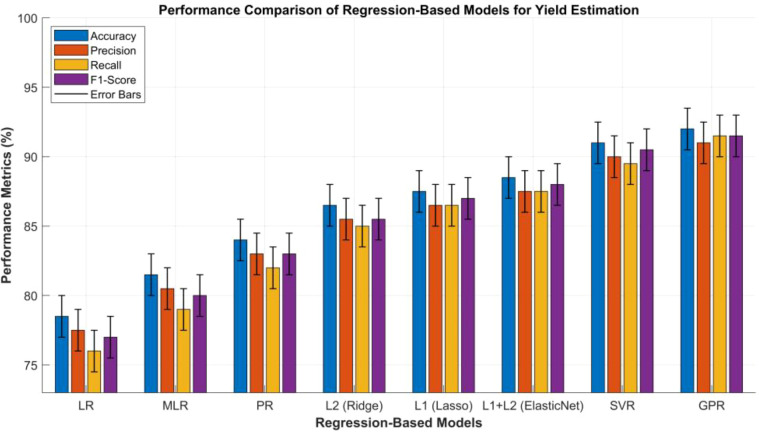
Comparative analysis of commonly employed regression-based techniques for yield estimation in crops and plants.

The results presented in **Figure 8** provide a comparative analysis of commonly employed regression-based techniques for crop and plant yield estimation. These results are not original experimental outputs; rather, they are extracted and compiled from multiple previously published studies reviewed in this work. These results collectively demonstrate a clear progression in performance from simpler models to more advanced and sophisticated regression approaches.

- LR achieves approximately 78% to 80% accuracy, indicating limited capability in modeling non-linear relationships.
- MLR and PR improve slightly, reaching 81% to 85% accuracy.
- Regularized models such as L1, L2, and Elastic Net (L1+L2) demonstrate enhanced stability and robustness, achieving 86% to 89% across all metrics.
- In contrast, advanced non-linear frameworks such as SVR and GPR exhibit superior predictive precision, achieving 91% to 95% accuracy.

The inclusion of error bars emphasizes the consistency of these models, with GPR demonstrating minimal variance and the highest robustness under complex, noisy, and irregular data conditions in the field/observed environment.

**Figure 9:**
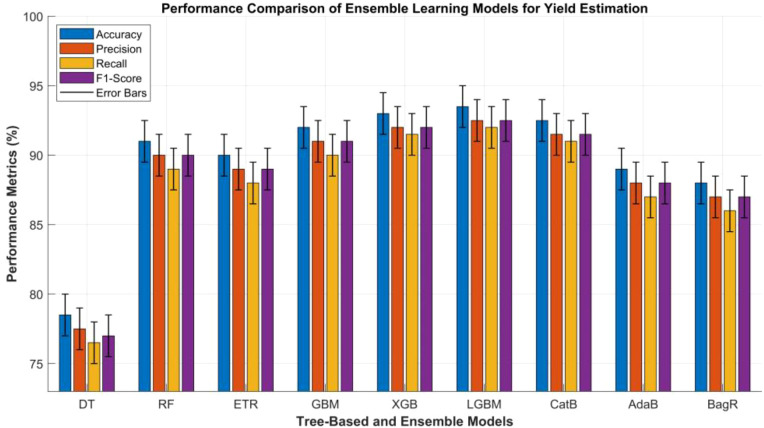
Comparative analysis of commonly employed ensemble and supervised-based learning models for yield estimation in crops and plants.

The results shown in **Figure 9** present a comparative analysis of commonly employed Ensemble and supervised-based Learning Models used for yield estimation in crops and plants. While a progressive improvement was observed in the results among regression-based models, the trend differed for ensemble-based approaches.

- Tree-based ensemble algorithms such as XGBoost, LightGBM, and CatBoost achieved the highest accuracy, ranging from 94% to 95%. Closer examination revealed well-balanced performance across all evaluation metrics. This consistency suggests enhanced generalization capability and greater robustness under varying conditions.
- GBMs and RF followed closely with 91% to 93% accuracy scores.
- DT showed inferior performance, with accuracy scores ranging between 78% and 80%, demonstrating its tendency toward overfitting.

The minimal error bars across models confirm their consistency in predictive stability across diverse agro-environmental datasets.

**Figure 10:**
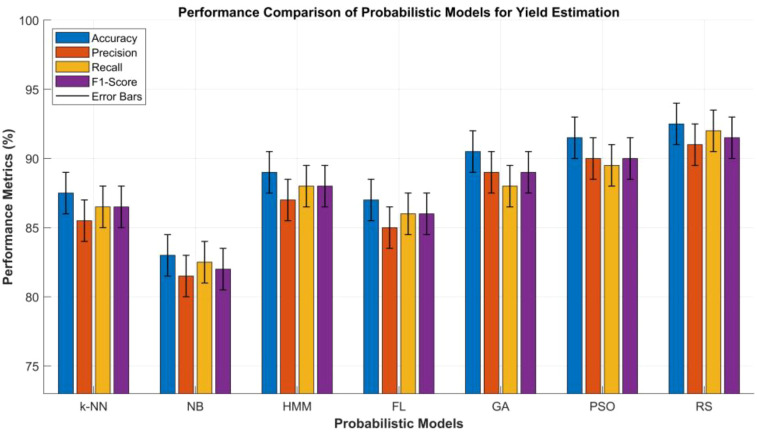
Comparative analysis of commonly employed probabilistic and supervised learning models along with optimization algorithms used for yield estimation in crops and plants.

The results shown in **Figure 10** present a comparative analysis of commonly employed Probabilistic, Supervised, and optimization-based models for yield estimation in crops and plants.

- RSM and PSO demonstrated superior predictive accuracy, achieving 92% to 93%, with balanced performance across all metrics, confirming their high adaptability to non-linear agro-ecological datasets.
- GA and HMM also maintained strong consistency, achieving accuracy between 89% and 91%.
- NB recorded the weakest results, with the accuracy score ranging from 82% to 84%, likely due to its simplistic independence assumptions.

The minimal overlap of error bars among top-performing models indicates statistical reliability and robustness.

**Figure 11:**
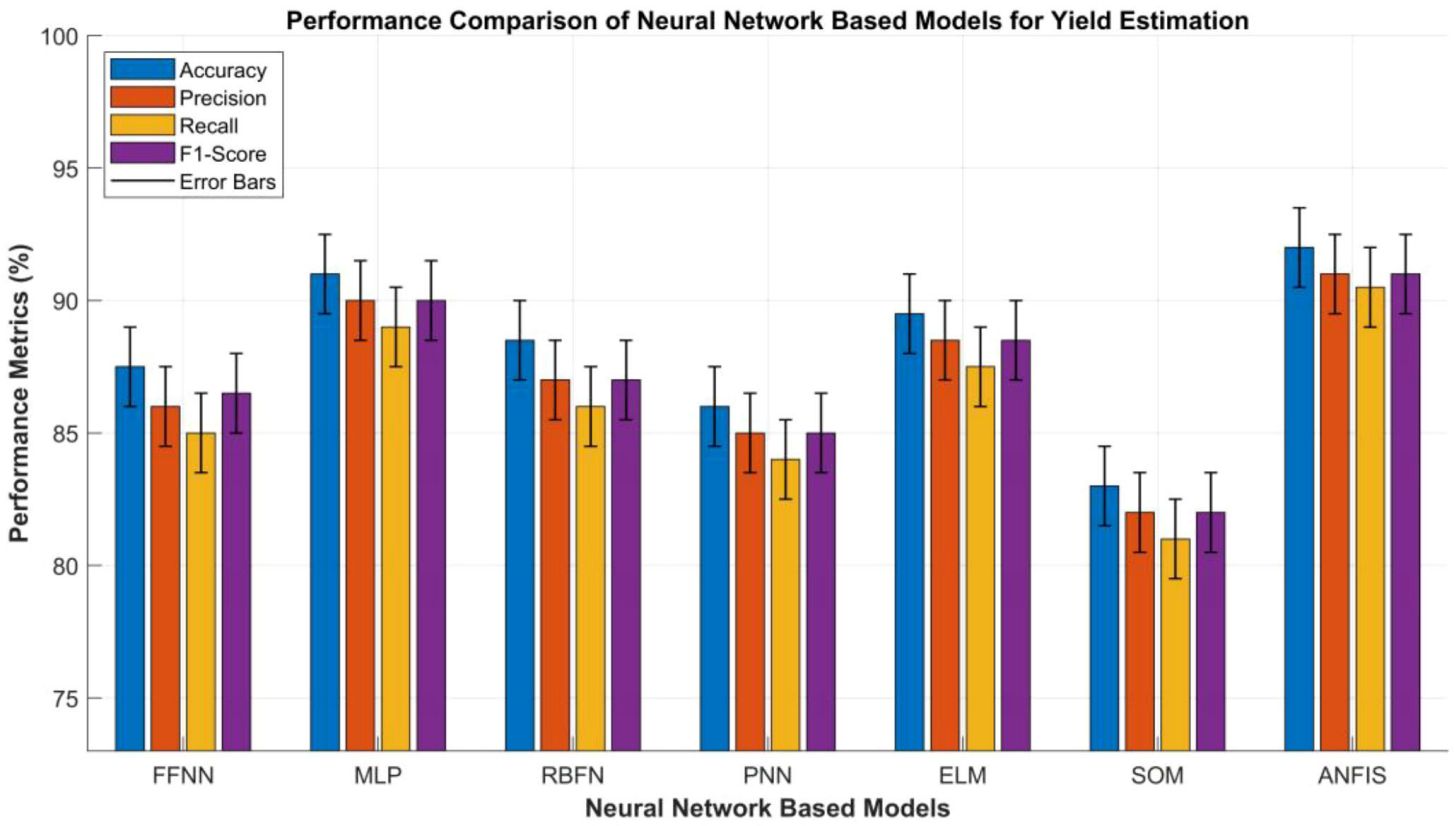
Comparative analysis of commonly employed neural network-based models for yield estimation in crops and plants.

The results shown in **Figure 11** present a comparative analysis of commonly employed neural network-based models for yield estimation in crops and plants.

- Among all models, ANFIS and MLP demonstrated superior performance, achieving approximately 92% to 94% accuracy, and highlighting their robust, learning capacity, and strong non-linear mapping between multisource input variables.
- ELM and RBFN showed competitive results, maintaining accuracy between 88% and 90%. These competitive results can be attributed to their efficient training and reduced computational complexity.
- In contrast, SOM and PNN exhibited lower performance, averaging between 82% and 86%. This low performance indicates their limited generalization ability and sensitivity to high-dimensional variability.

However, for the error bars, it can be assumed that the minimal overlap in top-performing models suggests statistical reliability and robustness.

**Figure 12:**
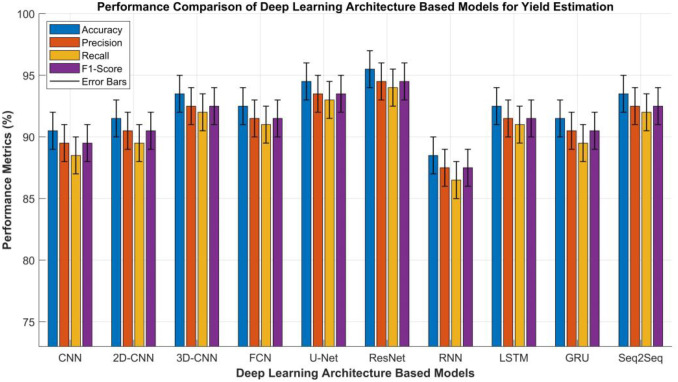
Comparative analysis of commonly employed deep learning-based models for yield estimation in crops and plants.

The results shown in **Figure 12** present a comparative analysis of commonly employed deep learning-based models for yield estimation in crops and plants.

- ResNet and U-Net architectures outperformed all others, achieving better average accuracy score of approximately 95% to 96%. This good performance indicates strong generalization capability and robustness against noise.
- In contrast, conventional CNNs exhibited relatively lower performance, with accuracy scores between 89% and 91%. These low accuracy scores reflect limited feature representation of power for complex spatiotemporal yield variations in the data.
- 3D-CNNs and FCNs showed noticeable improvements, achieving 92% to 94% accuracy by effectively capturing volumetric and contextual information.
- RNN-based models excelled in modeling temporal dependencies in sequential phenological data, achieving accuracies in the 91% to 94% range.

The inclusion of error bars enables readers to underscore the total variability, which can be due to stochastic initialization and training data heterogeneity.

Based on the collective findings obtained from various researchers and presented in the form of bar graphs in this section, it can be inferred that among regression-based approaches, GPR achieved superior predictive accuracy. The reason for this good performance is primarily due to its probabilistic inference mechanism for modeling complex non-linear relationships effectively. Ensemble learning methods, particularly CatBoost and LightGBM, outperformed all conventional models. This advantage stems from their effective utilization of gradient boosting frameworks combined with advanced regularization mechanisms, which enhance predictive accuracy and mitigate overfitting. This leveraging further helps it to handle heterogeneous agricultural data used for yield estimation. Within the probabilistic domain, RSM achieved competitive accuracy, reflecting its robustness in noisy environments. For NN architectures, ANFIS demonstrated superior adaptability by combining fuzzy logic with neural learning, achieving comparable precision. However, DL architectures, especially ResNet and Seq2Seq, achieved the highest predictive reliability due to their ability to capture spatiotemporal correlations and complex feature hierarchies. Overall, this comparison clearly establishes that while traditional and ensemble approaches remain computationally efficient, DL models deliver the most consistent and generalized performance. This output makes them the optimal choice for large-scale, data-intensive yield estimation tasks in precision agriculture for both crops-based and plant-based dataset scenarios.

However, all discussed results in this section were obtained using various algorithm/models and feature sets. For improved clarity and ease of interpretation, these features are systematically categorized in **Table 9**.

**Table 9:** Features of crop and plants used for yield estimation.

S.No	Category	Feature examples	Description/importance	Limitations	Suitable models	References
1	Spectral Reflectance & Vegetation Indices	NIR, NDVI, EVI, SAVI, GNDVI, NDRE,	Quantify canopy greenness, chlorophyll concentration, and photosynthetic activity to indicate crop vigor and biomass.	Saturation at high biomass, influenced by soil background and atmospheric noise.	LR, MLR, PR, SVR, GPR, RF, GBMs, XGBoost, LightGBM, CNNs, 2D CNN	(Kok et al., 2021; Piekutowska et al., 2021; Prasad et al., 2021; Yang et al., 2021; Ghosh et al., 2022b; Huber et al., 2022; Jha et al., 2022; Dhillon et al., 2023; Iniyan et al., 2023; Kulpanich et al., 2023; Lagrazon and Tan, 2023; Morales et al., 2023; Uribeetxebarria et al., 2023; Wang et al., 2025)
2	Thermal & Stress Indices	Canopy Temperature, CWSI, Thermal Vegetation Index	Detect water, temperature, and transpiration related to stress in crops.	Sensitive to environmental conditions, calibration required.	SVR, GPR, RF, ETR, GBM, LSTM, GRU	(Alibabaei et al., 2021; Kok et al., 2021; Prasad et al., 2021; Ghosh et al., 2022b; Huber et al., 2022; Dhillon et al., 2023; Kulpanich et al., 2023; Lagrazon and Tan, 2023; Mateo-Sanchis et al., 2023; Uribeetxebarria et al., 2023; Sudhamathi and Perumal, 2024; Hussein et al., 2025; Wang et al., 2025)
3	Canopy Structural Metrics	Canopy Height Model, LAI, Canopy Cover, Leaf Angle Distribution	Indicate plant structure, density, and biomass accumulation.	Require accurate 3D canopy reconstruction, prone to show errors under dense canopies.	RF, GBM, ETR, XGBoost, LightGBM, CNN, 3D CNN, U-Net	(Prasad et al., 2021; Yang et al., 2021; Huber et al., 2022; Nejad et al., 2022; Dhillon et al., 2023; Kulpanich et al., 2023; Uribeetxebarria et al., 2023; Buttar, 2024; Sudhamathi and Perumal, 2024; Wang et al., 2025)
4	3D Plant Architecture	Plant Height, Volume, Crown Width, Surface Roughness	Derived from LiDAR or photogrammetry to estimate growth and yield.	Computationally expensive and is also affected by terrain and wind distortion.	CNN, 3D CNN, FCN, ResNet, U-Net	(Yang et al., 2021; Nejad et al., 2022; Fathi et al., 2023; Buttar, 2024)
5	Texture & Spatial Pattern Features	Gray Level Co-Occurrence Matrix (GLCM) Contrast, Entropy, Homogeneity Correlation	Quantify spatial variability and canopy texture from images.	Dependent on image resolution and window size, Have the tendency to misrepresent mixed pixels.	CNN, 2D CNN, ResNet, ETR, CatBoost	(Yang et al., 2021; Fathi et al., 2023; Morales et al., 2023; Uribeetxebarria et al., 2023; Sudhamathi and Perumal, 2024)
6	Meteorological & Climatic Variables	Temperature (Max/Min/Avg), Rainfall, Humidity, Evapotranspiration	Represent environmental and energy balance factors influencing crop yield.	Sparse or missing station data, microclimate variability.	MLR, SVR, RF, GBM, LSTM, GRU, Seq2Seq	(Alibabaei et al., 2021; Kok et al., 2021; Piekutowska et al., 2021; Prasad et al., 2021; Huber et al., 2022; Dhillon et al., 2023; Kulpanich et al., 2023; Mateo-Sanchis et al., 2023; Uribeetxebarria et al., 2023; Hussein et al., 2025; Orduna-Cabrera et al., 2025; Wang et al., 2025)
7	Thermal Dynamics	Growing Degree Days (GDD), Cumulative Heat Units	Model crop phenological development stages tied to yield potential.	Crop specific data tuning required, ignores sudden temperature extremes.	SVR, RF, GBM, LSTM, GRU	(Alibabaei et al., 2021; Kok et al., 2021; Prasad et al., 2021; Huber et al., 2022; Dhillon et al., 2023; Kulpanich et al., 2023; Mateo-Sanchis et al., 2023; Uribeetxebarria et al., 2023; Hussein et al., 2025; Wang et al., 2025)
8	Soil Physical Properties	Moisture, Texture, pH, EC, Organic Matter, Bulk Density	Determine root zone conditions for water and nutrient transport.	High spatial heterogeneity and the data collection may also be labor intensive.	RF, GPR, SVR, GBM, FNN, MLP	(Kok et al., 2021; Prasad et al., 2021; Ghosh et al., 2022b; Huber et al., 2022; Tripathi et al., 2022; Dhillon et al., 2023; Kulpanich et al., 2023; Lagrazon and Tan, 2023; Uribeetxebarria et al., 2023; Chang et al., 2024; Wang et al., 2025)
9	Soil Chemical Properties	N, P, K, Ca, Mg, Fe concentrations	Indicate nutrient availability and fertility influencing productivity.	Requires periodic laboratory analysis, limited temporal coverage.	RF, GPR, ETR, SVR, MLP, ANFIS	(Kok et al., 2021; Prasad et al., 2021; Ghosh et al., 2022b; Tripathi et al., 2022; Dhillon et al., 2023; Lagrazon and Tan, 2023; Borse et al., 2025)
10	Topographic Features	Elevation, Slope Aspect	Control drainage, sunlight exposure, and erosion dynamics.	Static over time and is less significant in non-uniform plains.	RF, GBM, GPR, PSO	(Prasad et al., 2021; Ghosh et al., 2022b; Huber et al., 2022; Dhillon et al., 2023; Gong et al., 2023; Kulpanich et al., 2023; Lagrazon and Tan, 2023; Uribeetxebarria et al., 2023; Wang et al., 2025)

## 5 Conclusion

RS technology has significantly advanced agricultural monitoring and yield estimation practices. It has empowered the research community through data-driven analytical frameworks that support large-scale, non-invasive assessment methodologies for evaluating crop performance metrics. This advancement has helped improve the evaluation processes of plant physiological characteristics. To further improve yield estimation, researchers are continuously opting for several newer platforms and model-based approaches. These approaches leverage improved decision-support systems to more effectively detect stress factors and quantify their impact on biomass distribution patterns. The integration of multispectral, hyperspectral, LiDAR, radar, and thermal imaging with sophisticated modeling tools and ML techniques has further enhanced the precision and timeliness of yield forecasting. By analyzing all the benefits of RS in yield estimation, it can be concluded that it serves as a foundation for precise yield estimation in the modern era. However, it also has some limitations, which we have divided into four categories: (i) Environmental, (ii) Algorithmic, (iii) Hardware and Operational, and (iv) WSN-based limitations. Collectively, these factors undermine the ability of RS systems to accurately estimate crop yield, as they degrade data integrity and, more critically, reduce the reliability and precision of predictive outcomes. Limited accessibility to RS technologies remains a significant constraint in many practical scenarios. An analysis of studies integrating RS-based datasets with predictive algorithms reveals that DL architecture-based models consistently deliver superior performance in crop yield estimation. Based on this review, it can be concluded that the adoption of refined and accessible RS solutions is indispensable for supporting resilient and sustainable agricultural systems. The discussed advancements are expected to play a critical role in addressing the increasing global demand for food.

Advancements in UAV battery storage capacity are essential to effectively mitigate existing energy and power constraints. In parallel, improvements at the WSN level, particularly in the design of energy-efficient routing protocols, are necessary to enable data transmission between nodes with minimal energy consumption. There is a clear need to develop agriculture application-specific cybersecurity mechanisms to enhance the reliability, integrity, and security of data usage. In addition, establishing a global, researcher-accessible repository of manually collected yield-estimation datasets is essential for improving and validating models and algorithms. The integration of emerging wireless communication media in agricultural RS is essential, as conventional electromagnetic (EM) wave-based systems are inadequate in many scenarios due to signal degradation caused by crop and plant interference. Among these alternatives, Magneto-Inductive (MI) communication stands out for its robustness in complex agricultural environments, as most obstacles present are carbon-based and have no impact on MI signal propagation. To prolong the lifetime of a working equipment in an RS-based WSN environment, it is essential to consider the development of monitoring equipment capable of supporting Simultaneous Wireless Information and Power Transfer (SWIPT).

## Glossary

2D-CNNs: Two-Dimensional CNNs
3D: Three-Dimensional
3D-CNNs: Three-Dimensional CNNs
ANFIS: Adaptive Neuro-Fuzzy Inference System
ANNs: Artificial Neural Networks
APSIM: Agricultural Production Systems Simulator
AES: Advanced Encryption Standard
BRDF: Bidirectional Reflectance Distribution Function
BLE: Bluetooth Low Energy
Ca: Calcium
CatBoost: Categorical Boosting
CNNs: Convolutional Neural Networks
CWSI: Crop Water Stress Index
DDoS: Distributed Denial –of Service
DL: Deep Learning
DoS: Denial Of Services
DSSAT: Decision Support System for Agro-Technology Transfer
DT: Decision Trees
DTLS: Datagram Transport Layer Security
ELM: Extreme Learning Machine
ETR: Extra Trees Regressor
EVI: Enhanced Vegetation Index
ECC: Error Correction Code
FCNs: Fully Convolutional Networks
FDI: False Data Injection
Fe: Iron
GBM’s: Gradient Boosting Machines
GDD: Growing Degree Days
GLCM: Gray-Level Co-Occurrence Matrix
GNDVI: Green Normalized Difference Vegetation Index
GPR: Gaussian Process Regression
GPS: Global Positioning Systems
GRUs: Gated Recurrent Units
HSV: Hue, Saturation, Value
ICA: Independent Component Analysis
IoT: Internet of Things
K: Potassium
K-NNs: K-Nearest Neighbors
L1: Ridge Regression
L2: Lasso Regression
LAI: Leaf Area Index
LBP: Local Binary Pattern
LiDAR: Light Detection and Ranging
LightGBM: Light Gradient Boosting Machine
LR: Linear Regression
LSTM: Long Short-Term Memory
LoRa: Long Range
MAS: Manned Aerial Systems
Mg: Magnesium
MITM: Man-in-the-Middle
ML: Machine Learning
MLR: Multiple Linear Regression
N: Nitrogen
NDRE: Normalized Difference Red Edge
NDVI: Normalized Difference Vegetation Index
NIR: Near Infrared
P: Phosphorous
PAM: Pulse Amplitude Modulation
PCA: Principal Component Analysis
pH: Potential of Hydrogen
PNNs: Probabilistic Neural Networks
PR: Polynomial Regression
PRI: Photochemical Reflectance Index
PSO: Particle Swarm Optimization
RBFN: Radial Basis Function Network
R-CNNs: Region-Based Convolutional Neural Networks
Res-Net: Residual Neural Networks
RF: Random Forest
RGB: Red, Green, Blue
RL: Reinforcement Learning
RNNs: Recurrent Neural Networks
RS: Remote Sensing
RSM: Random Subspace Method
SAR: Synthetic Aperture Radar
SAVI: Soil-Adjusted Vegetation Index
Seq2Seq: Sequence-to-Sequence
SNR: Signal-to-Noise Ratio
SOM: Self-Organizing Map
SPAD: Soil Plant Analysis Development
SQL: Structured Query Language
SVM: Support Vector Machines
SVR: Support Vector Regression
SWIPT: Simultaneous Wireless Information and Power Transfer
TIR: Thermal Infrared
TOF: Time of Flight
TLS: Transport Layer Security
UAVs: Unmanned Aerial Vehicles
VIs: Vegetation Indices
VPN: Virtual Private Network
WOFOST: World Food Studies
WSNs: Wireless Sensor Networks
Wi-Fi: Wireless Fidelity
XGBoost: Extreme Gradient Boosting
XML: Extensible Markup Language
YOLO: You Only Look Once
ZigBee: Zonal Intercommunication Global Standard
MAC: Media Access Control
MFA: Multi-Factor Authentication
WAF: Web Application Firewall
PLC: Programmable Logic Controller
OTA: One-Time Authentication
API: Application Programming Interface.

## References

[B1] Abayechaw D. (2021). Review on decision support system for agrotechnology transfer (DSSAT) model. Inf. Syst. 10, 126–133. doi: 10.11648/j.ijiis.20211006.13

[B2] Abdelbaki A. Udelhoven T. (2022). A review of hybrid approaches for quantitative assessment of crop traits using optical remote sensing: research trends and future directions. Remote Sens. 14, 3515. doi: 10.3390/rs14153515, PMID: 41725453

[B3] Abdul-Jabbar T. Ziboon A. Albayati M. (2023). “ Crop yield estimation using different remote sensing data: literature review,” in IOP conference series: earth and environmental science, vol. 012004. ( IOP Publishing). Bristol, England.

[B4] Abreu Júnior C. Martins G. D. Xavier L. C. M. Vieira B. S. Gallis R. Fraga Junior E. F. . (2022). Estimating coffee plant yield based on multispectral images and machine learning models. Agronomy 12, 3195. doi: 10.3390/agronomy12123195, PMID: 41725453

[B5] Adewusi A. O. Chiekezie N. R. Eyo-Udo N. L. (2022). The role of AI in enhancing cybersecurity for smart farms. World J. Advanced Res. Rev. 15, 501–512. doi: 10.30574/wjarr.2022.15.3.0889

[B6] Aggarwal K. Reddy G. S. Makala R. Srihari T. Sharma N. Singh C. (2024). Studies on energy efficient techniques for agricultural monitoring by wireless sensor networks. Comput. Electrical Eng. 113, 109052. doi: 10.1016/j.compeleceng.2023.109052, PMID: 41834798

[B7] Ahmed A. M. Sharma E. Jui S. J. J. Deo R. C. Nguyen-Huy T. Ali M. (2022). Kernel ridge regression hybrid method for wheat yield prediction with satellite-derived predictors. Remote Sens. 14, 1136. doi: 10.3390/rs14051136, PMID: 41725453

[B8] Akanksha E. Sharma N. Gulati K. (2021). “ OPNN: optimized probabilistic neural network based automatic detection of maize plant disease detection,” in 2021 6th international conference on inventive computation technologies (ICICT) ( IEEE), New York City, USA, 1322–1328.

[B9] Alibabaei K. Gaspar P. D. Lima T. M. (2021). Crop yield estimation using deep learning based on climate big data and irrigation scheduling. Energies 14, 3004. doi: 10.3390/en14113004, PMID: 41725453

[B10] Alkhudaydi T. de la Lglesia B. (2022). Counting spikelets from infield wheat crop images using fully convolutional networks. Neural Computing Appl. 34, 17539–17560. doi: 10.1007/s00521-022-07392-1, PMID: 41835834

[B11] Asner G. P. Ustin S. L. Townsend P. Martin R. E. (2024). “ Forest biophysical and biochemical properties from hyperspectral and LiDAR remote sensing,” in Remote sensing handbook, volume IV ( CRC Press), Milton Park, Abingdon-on-Thames in the United Kingdom, 96–124.

[B12] Awate P. Nagne A. (2023). “ Enhanced vegetation cover assessment using sentinel-2A: A unified perspective on NDVI, SAVI, and GNDVI,” in International conference on information science and applications ( Springer), London (global), Berlin (corporate), New York (sales), 257–271.

[B13] Barreto L. Amaral A. (2018). “ Smart farming: Cyber security challenges,” in 2018 international conference on intelligent systems (IS) ( IEEE), New York City, USA, 870–876.

[B14] Basso M. Stocchero D. Ventura Bayan Henriques R. Vian A. L. Bredemeier C. Konzen A. A. . (2019). Proposal for an embedded system architecture using a GNDVI algorithm to support UAV-based agrochemical spraying. Sensors 19, 5397. doi: 10.3390/s19245397, PMID: 31817832 PMC6960772

[B15] Battilani A. (2014). Limited access to resources: challenges or opportunities? In: ISHS Acta Horticulturae 1081: XIII International Symposium on Processing Tomato. International Society for Horticultural Science, Leuven, Belgium, 1081, 27–40.

[B16] Bi L. Hu G. (2021). A genetic algorithm-assisted deep learning approach for crop yield prediction. soft Computing 25, 10617–10628. doi: 10.1007/s00500-021-05995-9, PMID: 41835834

[B17] Bishop J. Garratt M. P. Nakagawa S. (2022). Animal pollination increases stability of crop yield across spatial scales. Ecol. Lett. 25, 2034–2047. doi: 10.1111/ele.14069, PMID: 35843226 PMC9544623

[B18] Boros A. Szólik E. Desalegn G. Tőzsér D. J. A. (2024). A systematic review of opportunities and limitations of innovative practices in sustainable agriculture. Agronomy mdpi 15, 76. doi: 10.3390/agronomy15010076, PMID: 41725453

[B19] Borse K. Agnihotri P. Jothimani M. Jena R. K. (2025). Adaptive neuro fuzzy inference system based multicrop yield prediction in the semi arid region of India. Sci. Rep. 15, 18787. doi: 10.1038/s41598-025-03334-8, PMID: 40442185 PMC12123030

[B20] Bustos-Korts D. Boer M. P. Layton J. Gehringer A. Tang T. Wehrens R. . (2022). Identification of environment types and adaptation zones with self-organizing maps; applications to sunflower multi-environment data in Europe. Theor. Appl. Genet. 135, 2059–2082. doi: 10.1007/s00122-022-04098-9, PMID: 35524815 PMC9205840

[B21] Buttar P. K. (2024). Satellite imagery analysis for crop type segmentation using U-net architecture. Proc. Comput. Sci. 235, 3418–3427. doi: 10.1016/j.procs.2024.04.322, PMID: 41834798

[B22] Cao Y. Sun G. Yuan Y. Chen L. (2025). Small-sample cucumber disease identification based on multimodal self-supervised learning. Crop Prot. 188, 107006. doi: 10.1016/j.cropro.2024.107006, PMID: 41834798

[B23] Chandana C. Parthasarathy G. (2022). Efficient machine learning regression algorithm using naïve Bayes classifier for crop yield prediction and optimal utilization of fertilizer. Int. J. Performability Eng. 18, 47. doi: 10.23940/ijpe.22.01.p6.4755

[B24] Chang N. A. K. Dey S. Das D. K. (2024). Nested sequential feed-forward neural network: A cumulative model for crop yield prediction. Comput. Electron. Agric. 227, 109562. doi: 10.1016/j.compag.2024.109562, PMID: 41834798

[B25] Chen J. Zhou J. Li Q. Li H. Xia Y. Jackson R. . (2023). CropQuant-Air: An AI-powered system to enable phenotypic analysis of yield-and performance-related traits using wheat canopy imagery collected by low-cost drones. Front. Plant Sci. 14, 1219983. doi: 10.3389/fpls.2023.1219983, PMID: 37404534 PMC10316027

[B26] Chen Y. Shi Y. Wang Z. Huang L. (2016). Connectivity of wireless sensor networks for plant growth in greenhouse. Int. J. Agric. Biol. Eng. 9, 89–98. doi: 10.3965/j.ijabe.201606901.1314

[B27] Cheng E. Zhang B. Peng D. Zhong L. Yu L. Liu Y. . (2022). Wheat yield estimation using remote sensing data based on machine learning approaches. Front. Plant Sci. 13, 1090970. doi: 10.3389/fpls.2022.1090970, PMID: 36618627 PMC9816798

[B28] Cheng H. Li H. Lian J. (2024). UAV-based Anomaly Detection via a novel Spatial-Temporal Transformer for Precision Agriculture. Computer aided design and applications 13, 298–310. doi: 10.14733/cadaps.2024.S13.298-310

[B29] Clement M. (2025). Vision transformer-based systems for crop disease detection and monitoring in precision agriculture.

[B30] Cunha I. A. Baptista G. M. Prudente V. H. R. Melo D. D. Amaral L. R. (2024). Integration of optical and synthetic aperture radar data with different synthetic aperture radar image processing techniques and development stages to improve soybean yield prediction. Agriculture 14, 2032. doi: 10.3390/agriculture14112032, PMID: 41725453

[B31] Debnath S. Paul M. Debnath T. (2023). Applications of LiDAR in agriculture and future research directions. J. Imaging 9, 57. doi: 10.3390/jimaging9030057, PMID: 36976108 PMC10052112

[B32] Demestichas K. Peppes N. Alexakis T. (2020). Survey on security threats in agricultural IoT and smart farming. sensors 20, 6458. doi: 10.3390/s20226458, PMID: 33198160 PMC7697696

[B33] Dhillon M. S. Dahms T. Kuebert-Flock C. Rummler T. Arnault J. Steffan-Dewenter I. . (2023). Integrating random forest and crop modeling improves the crop yield prediction of winter wheat and oil seed rape. Front. Remote Sens. 3, 1010978. doi: 10.3389/frsen.2022.1010978, PMID: 41835472

[B34] Dsouza R. P. Babu G. S. (2024). Integrating decision tree and KNN hybrid algorithm approach for enhancing agricultural yield prediction. Comput. Integrated Manufacturing Syst. 30, 15–32.

[B35] El-Ghamry A. Darwish A. Hassanien A. E. (2023). An optimized CNN-based intrusion detection system for reducing risks in smart farming. Internet Things 22, 100709. doi: 10.1016/j.iot.2023.100709, PMID: 41834798

[B36] Faizan G. S. Khilar R. (2025). “ Improving accuracy for crop yield prediction using Adaboost algorithm comparison with decision tree,” in AIP conference proceedings, vol. 020113. ( AIP Publishing LLC), 1305 Walt Whitman Road Suite 110 Melville, NY 11747-4300, USA

[B37] Falcioni R. Santos G. Crusiol L. G. T. Antunes W. C. Chicati M. L. Oliveira R. . (2023). Non– invasive assessment, classification, and prediction of biophysical parameters using reflectance hyperspectroscopy. Plants mdpi 12, 2526. doi: 10.3390/plants12132526, PMID: 37447089 PMC10347113

[B38] Farooq A. Farooq N. Akbar H. Hassan Z. U. Gheewala S. H. (2023). A critical review of climate change impact at a global scale on cereal crop production. Agronomy 13, 162. doi: 10.3390/agronomy13010162, PMID: 41725453

[B39] Fathi M. Shah-Hosseini R. Moghimi A. (2023). 3D-ResNet-BiLSTM model: a deep learning model for county-level soybean yield prediction with time-series Sentinel-1, Sentinel-2 imagery, and Daymet data. Remote Sens. 15, 5551. doi: 10.3390/rs15235551, PMID: 41725453

[B40] Fei S. Hassan M. A. Xiao Y. Su X. Chen Z. Cheng Q. . (2023). UAV-based multi-sensor data fusion and machine learning algorithm for yield prediction in wheat. Precis. Agric. 24, 187–212. doi: 10.1007/s11119-022-09938-8, PMID: 35967193 PMC9362526

[B41] Ferencz C. Bognár P. Lichtenberger J. Hamar D. Tarcsai G. Timár G. . (2004). Crop yield estimation by satellite remote sensing. Int. J. Remote Sens. 25, 4113–4149. doi: 10.1080/01431160410001698870, PMID: 41799851

[B42] Filippi P. Han S. Y. Bishop T. F. (2025). On crop yield modelling, predicting, and forecasting and addressing the common issues in published studies. Precis. Agric. 26, 8. doi: 10.1007/s11119-024-10212-2, PMID: 41835834

[B43] Fu H. Wang C. Cui G. She W. Zhao L. (2021). Ramie yield estimation based on UAV RGB images. Sensors 21, 669. doi: 10.3390/s21020669, PMID: 33477949 PMC7833380

[B44] Gade S. A. Madolli M. J. García-Caparrós P. Ullah H. Cha-um S. Datta A. . (2025). Advancements in UAV remote sensing for agricultural yield estimation: A systematic comprehensive review of platforms, sensors, and data analytics. Remote Sens. Applications: Soc. Environ. 37, 101418. doi: 10.1016/j.rsase.2024.101418, PMID: 41834798

[B45] García Cárdenas D. A. Ramón Valencia J. A. Alzate Velásquez D. F. Palacios Gonzalez J. R. (2018). “ Dynamics of the indices NDVI and GNDVI in a rice growing in its reproduction phase from multi-spectral aerial images taken by drones,” in International conference of ICT for adapting agriculture to climate change ( Springer), London (global), Berlin (corporate), New York (sales), 106–119.

[B46] Gatkal N. Dhar T. Prasad A. Prajwal R. Santosh J.B. Jyoti B. . (2024). Development of a user-friendly automatic ground-based imaging platform for precise estimation of plant phenotypes in field crops. J. Field Robotics 41, 2355–2372. doi: 10.1002/rob.22254, PMID: 41823357

[B47] Gavasso-Rita Y. L. Papalexiou S. M. Li Y. Elshorbagy A. Li Z. Schuster-Wallace C. (2024). Crop models and their use in assessing crop production and food security: A review. Food Energy Secur. 13, e503. doi: 10.1002/fes3.503, PMID: 41823357

[B48] George N. A. Cuamba H. Lundy M. E. Bennett S. J. (2024). Evaluating the Agricultural Production Systems sIMulator (APSIM) wheat module for California. Crop Pasture Sci. 75. doi: 10.1071/CP23046, PMID: 41161682

[B49] Ghochanian Haghverdi E. Yaghoobzadeh M. Moghri Friz A. Khorashadizadeh O. Javadi H. (2024). Impact of vapor pressure deficit on saffron yield and its prediction using artificial intelligence algorithms. J. Saffron Res. 12, 227–240.

[B50] Ghosh S. S. Dey S. Bhogapurapu N. Homayouni S. Bhattacharya A. McNairn H. (2022b). Gaussian process regression model for crop biophysical parameter retrieval from multi-polarized C-band SAR data. Remote Sens. 14, 934. doi: 10.3390/rs14040934, PMID: 41725453

[B51] Ghosh P. Mandal D. Wilfling S. Hollberg J. Bargiel D. Bhattacharya A. (2022a). Synergy of optical and synthetic aperture radar data for early-stage crop yield estimation: a case study over a state of Germany. Geocarto Int. 37, 10743–10766. doi: 10.1080/10106049.2022.2039306, PMID: 41799851

[B52] Goel R. K. Yadav C. S. Vishnoi S. Rastogi R. (2021). Smart agriculture–Urgent need of the day in developing countries. Sustain. Computing: Inf. Syst. 30, 100512. doi: 10.1016/j.suscom.2021.100512, PMID: 41834798

[B53] Gong L. Yu M. Kollias S. (2023). Optimizing crop yield and reducing energy consumption in greenhouse control using PSO-MPC algorithm. Algorithms 16, 243. doi: 10.3390/a16050243, PMID: 41725453

[B54] Grassini P. van Bussel L. G. Van Wart J. Wolf J. Claessens L. Yang H. . (2015). How good is good enough? Data requirements for reliable crop yield simulations and yield-gap analysis. Field Crops Res. 177, 49–63. doi: 10.1016/j.fcr.2015.03.004, PMID: 41834798

[B55] Gulshan I. Javaid A. Javed Z. Nawaz S. (2024). Exploring the factors related to the yield of sunflower crop: an application of robust and ridge regression analysis. Russian Law J. 12, 826–838.

[B56] Gupta M. Abdelsalam M. Khorsandroo S. Mittal S. (2020). Security and privacy in smart farming: Challenges and opportunities. IEEE Access 8, 34564–34584. doi: 10.1109/ACCESS.2020.2975142, PMID: 41116384

[B57] Hadria R. Duchemin B. Lahrouni A. Khabba S. Er-Raki S. Dedieu G. . (2006). Monitoring of irrigated wheat in a semi-arid climate using crop modelling and remote sensing data: Impact of satellite revisit time frequency. Int. J. Remote Sens. 27, 1093–1117. doi: 10.1080/01431160500382980, PMID: 41799851

[B58] Hazra S. Karforma S. Bandyopadhyay A. Chakraborty S. Chakraborty D. (2023). Prediction of crop yield using machine learning approaches for agricultural data. Authorea Preprints. doi: 10.36227/techrxiv.23694867.v1

[B59] Hu G. You F. (2022). Renewable energy-powered semi-closed greenhouse for sustainable crop production using model predictive control and machine learning for energy management. Renewable Sustain. Energy Rev. 168, 112790. doi: 10.1016/j.rser.2022.112790, PMID: 41834798

[B60] Huang L. Chen J. Li H. Huang Y. She K. Hao K. (2024). Excellent tomato detector based on pruning and distillation to balance accuracy and lightweight. Comput. Electron. Agric. 227, 109520. doi: 10.1016/j.compag.2024.109520, PMID: 41834798

[B61] Huber F. Yushchenko A. Stratmann B. Steinhage V. (2022). Extreme Gradient Boosting for yield estimation compared with Deep Learning approaches. Comput. Electron. Agric. 202, 107346. doi: 10.1016/j.compag.2022.107346, PMID: 41834798

[B62] Hussein L. Johri P. Priya A. A. Ramya R. Karpagam J. Devendran A. (2025). “ Crop yield forecasting using bidirectional gated recurrent unit (Bi-GRU) networks,” in 2025 international conference on automation and computation (AUTOCOM) ( IEEE), New York City, USA, 224–228.

[B63] Ilyas Q. M. Ahmad M. Mehmood A. (2023). Automated estimation of crop yield using artificial intelligence and remote sensing technologies. Bioengineering 10, 125. doi: 10.3390/bioengineering10020125, PMID: 36829619 PMC9952812

[B64] Ingole V. S. Kshirsagar U. A. Singh V. Yadav M. V. Krishna B. Kumar R. (2024). A hybrid model for soybean yield prediction integrating convolutional neural networks, recurrent neural networks, and graph convolutional networks. Computation 13, 4. doi: 10.3390/computation13010004, PMID: 41725453

[B65] Iniyan S. Varma V. A. Naidu C. T. (2023). Crop yield prediction using machine learning techniques. Adv. Eng. Software 175, 103326. doi: 10.1016/j.advengsoft.2022.103326, PMID: 41834798

[B66] Isinkaye F. O. Olusanya M. O. Akinyelu A. A. (2025). A multi-class hybrid variational autoencoder and vision transformer model for enhanced plant disease identification. Intelligent Syst. Appl. 26, 200490. doi: 10.1016/j.iswa.2025.200490, PMID: 41834798

[B67] Islam A. Ghose M. (2025). GSAgri: Green and Secure Agriculture through efficient task offloading and scheduling under IoT-enabled energy-harvesting multi-access edge computing framework. Expert Syst. Appl. 284, 127814. doi: 10.1016/j.eswa.2025.127814, PMID: 41834798

[B68] Jabed M. A. Murad M. A. A. (2024). Crop yield prediction in agriculture: A comprehensive review of machine learning and deep learning approaches, with insights for future research and sustainability. Heliyon 10. doi: 10.1016/j.heliyon.2024.e40836, PMID: 39720079 PMC11667600

[B69] Jácome Galarza L. Realpe M. Viñán-Ludeña M. S. Calderón M. F. Jaramillo S. (2025). AgriTransformer: A transformer-based model with attention mechanisms for enhanced multimodal crop yield prediction. Electronics 14, 2466. doi: 10.3390/electronics14122466, PMID: 41725453

[B70] Jha S. K. Patil V. C. Rekha B. Virnodkar S. S. Bartalev S. A. Plotnikov D. . (2022). Sugarcane yield prediction using vegetation indices in Northern Karnataka, India. Univ J. Agric. Res. 10, 699–721. doi: 10.13189/ujar.2022.100611

[B71] Ji Z. Pan Y. Zhu X. Wang J. Li Q. J. S. (2021). Prediction of crop yield using phenological information extracted from remote sensing vegetation index. Sensors mdpi 21, 1406. doi: 10.3390/s21041406, PMID: 33671356 PMC7922106

[B72] Joshi A. Pradhan B. Gite S. Chakraborty S. (2023). Remote-sensing data and deep-learning techniques in crop mapping and yield prediction: A systematic review. Remote Sens. 15, 2014. doi: 10.3390/rs15082014, PMID: 41725453

[B73] Kalichkin V. Alsova O. Maksimovich K. Y. (2021). “ Application of the decision tree method for predicting the yield of spring wheat,” in IOP conference series: earth and environmental science, vol. 032042. ( IOP Publishing), Bristol, England.

[B74] Kamal S. Sharma P. Gupta P. Siddiqui M. K. Singh A. Dutt A. (2025). DVTXAI: a novel deep vision transformer with an explainable AI-based framework and its application in agriculture. J. Supercomputing 81, 280. doi: 10.1007/s11227-024-06494-y, PMID: 41835834

[B75] Kamilaris A. Kartakoullis A. Prenafeta-Boldú F. X. (2017). A review on the practice of big data analysis in agriculture. Comput. Electron. Agric. 143, 23–37. doi: 10.1016/j.compag.2017.09.037, PMID: 41834798

[B76] Katimbo A. Rudnick D. R. DeJonge K. C. Lo T. H. Qiao X. Franz T. E. . (2022). Crop water stress index computation approaches and their sensitivity to soil water dynamics. Agric. Water Manage. 266, 107575. doi: 10.1016/j.agwat.2022.107575, PMID: 41834798

[B77] Kaya Y. Polat N. (2023). A linear approach for wheat yield prediction by using different spectral vegetation indices. Int. J. Eng. Geosciences 8, 52–62. doi: 10.26833/ijeg.1035037

[B78] Khanal S. Kc K. Fulton J. P. Shearer S. Ozkan E. (2020). Remote sensing in agriculture—accomplishments, limitations, and opportunities. Remote Sens. 12, 3783. doi: 10.3390/rs12223783, PMID: 41725453

[B79] Kok Z. H. Shariff A. R. M. Alfatni M. S. M. Khairunniza-Bejo S. (2021). Support vector machine in precision agriculture: a review. Comput. Electron. Agric. 191, 106546. doi: 10.1016/j.compag.2021.106546, PMID: 41834798

[B80] Kordi F. Yousefi H. (2022). Crop classification based on phenology information by using time series of optical and synthetic-aperture radar images. Remote Sens. Applications: Soc. Environ. 27, 100812. doi: 10.1016/j.rsase.2022.100812, PMID: 41834798

[B81] Kosmowski F. Chamberlin J. Ayalew H. Sida T. Abay K. Craufurd P. (2021). How accurate are yield estimates from crop cuts? Evidence from smallholder maize farms in Ethiopia. Food Policy 102, 102122. doi: 10.1016/j.foodpol.2021.102122, PMID: 34898811 PMC8639447

[B82] Kulpanich N. Worachairungreung M. Thanakunwutthirot K. Chaiboonrueang P. (2023). The application of unmanned aerial vehicles (UAVs) and extreme gradient boosting (XGBoost) to crop yield estimation: A case study of don tum district, Nakhon Pathom, Thailand. Int. J. Geoinformatics 19, 65–77. doi: 10.52939/ijg.v19i2.2569

[B83] Kuzman B. Petković B. Denić N. Petković D. Ćirković B. Stojanović J. . (2021). Estimation of optimal fertilizers for optimal crop yield by adaptive neuro fuzzy logic. Rhizosphere 18, 100358. doi: 10.1016/j.rhisph.2021.100358, PMID: 41834798

[B84] Lagrazon P. G. G. Tan J. B. (2023). “ Predicting crop yield in quezon province, Philippines using gaussian process regression: A data-driven approach for agriculture sustainability,” in 2023 international conference on modeling & E-information research, artificial learning and digital applications (ICMERALDA) ( IEEE), New York City, USA, 7–12.

[B85] Lee W.-S. Alchanatis V. Yang C. Hirafuji M. Moshou D. Li C. (2010). Sensing technologies for precision specialty crop production. Comput. Electron. Agric. 74, 2–33. doi: 10.1016/j.compag.2010.08.005, PMID: 41834798

[B86] Lei L. Yang Q. Yang L. Shen T. Wang R. Fu C. (2024). Deep learning implementation of image segmentation in agricultural applications: a comprehensive review. Artif. Intell. Rev. 57, 149. doi: 10.1007/s10462-024-10775-6, PMID: 41835834

[B87] Li X. Tan J. Wang X. Han G. Qian Z. Li H. . (2024). Responses of spring wheat yield and growth period to different future climate change models in the yellow river irrigation area based on CMIP6 and WOFOST models. Agric. For. Meteorol 353, 110071. doi: 10.1016/j.agrformet.2024.110071, PMID: 41834798

[B88] Li J. Wang R. Zhang M. Wang X. Yan Y. Sun X. . (2023). A method for estimating alfalfa (Medicago sativa L.) forage yield based on remote sensing data. Agronomy 13. doi: 10.3390/agronomy13102597, PMID: 41725453

[B89] Li J. Wu W. Zhao C. Bai X. Dong L. Tan Y. . (2025a). Effects of solar elevation angle on the visible light vegetation index of a cotton field when extracted from the UAV. Sci. Rep. 15, 18497. doi: 10.1038/s41598-025-00992-6, PMID: 40425651 PMC12117064

[B90] Li Y. Ye L. Zhang Y. Zhou L. Chen Q. (2025b). Lightweight grading segmentation network for powdery mildew based on model pruning and knowledge distillation. Smart Agric. Technol. 101319. doi: 10.1016/j.atech.2025.101319, PMID: 41834798

[B91] Lillo-Saavedra M. Espinoza-Salgado A. García-Pedrero A. Souto C. Holzapfel E. Gonzalo-Martín C. . (2022). Early estimation of tomato yield by decision tree ensembles. Agriculture 12, 1655. doi: 10.3390/agriculture12101655, PMID: 41725453

[B92] Lin F. Crawford S. Guillot K. Zhang Y. Chen Y. Yuan X. . (2023). “ Mmst-vit: Climate change-aware crop yield prediction via multi-modal spatial-temporal vision transformer,” in Proceedings of the IEEE/CVF international conference on computer vision, IEEE, New York City, U.S. 5774–5784.

[B93] Linsner S. Varma R. Reuter C. (2019). “ Vulnerability assessment in the smart farming infrastructure through cyberattacks. In: GIL Annual Conference 2019, P287 - 39th GIL Annual Conference 2019, Bonn, Germany, ( Gesellschaft für Informatik eV).

[B94] Longchamps L. Tisseyre B. Taylor J. Sagoo L. Momin A. Fountas S. . (2022). Yield sensing technologies for perennial and annual horticultural crops: a review. Precis. Agric. 23, 2407–2448. doi: 10.1007/s11119-022-09906-2, PMID: 41835834

[B95] Lu Y. Lu X. Zheng L. Sun M. Chen S. Chen B. . (2024). Application of multimodal transformer model in intelligent agricultural disease detection and question-answering systems. plants 13, 972. doi: 10.3390/plants13070972, PMID: 38611501 PMC11013167

[B96] Lungu O. N. Chabala L. M. Shepande C. (2020). Satellite-based crop monitoring and yield estimation—a review. J. Agric. Sci. 13, 180. doi: 10.5539/jas.v13n1p180

[B97] Luo S. Jiang X. Yang K. Li Y. Fang S. (2022). Multispectral remote sensing for accurate acquisition of rice phenotypes: Impacts of radiometric calibration and unmanned aerial vehicle flying altitudes. Front. Plant Sci. 13, 958106. doi: 10.3389/fpls.2022.958106, PMID: 36035659 PMC9401905

[B98] Ma C. Liu M. Ding F. Li C. Cui Y. Chen W. . (2022). Wheat growth monitoring and yield estimation based on remote sensing data assimilation into the SAFY crop growth model. Sci. Rep. 12, 5473. doi: 10.1038/s41598-022-09535-9, PMID: 35361910 PMC8971471

[B99] Ma Y. Zhang Z. Kang Y. Özdoğan M. (2021). Corn yield prediction and uncertainty analysis based on remotely sensed variables using a Bayesian neural network approach. Remote Sens. Environ. 259, 112408. doi: 10.1016/j.rse.2021.112408, PMID: 41834798

[B100] Majumdar P. Mitra S. Bhattacharya D. (2021). IoT for promoting agriculture 4.0: a review from the perspective of weather monitoring, yield prediction, security of WSN protocols, and hardware cost analysis. J. Biosyst. Eng. 46, 440–461. doi: 10.1007/s42853-021-00118-6, PMID: 41835834

[B101] Manoj T. Makkithaya K. Narendra V. (2023). A trusted IoT data sharing and secure oracle based access for agricultural production risk management. Comput. Electron. Agric. 204, 107544. doi: 10.1016/j.compag.2022.107544, PMID: 41834798

[B102] Mateo-Sanchis A. Adsuara J. E. Piles M. Munoz-Marí J. Perez-Suay A. Camps-Valls G. (2023). Interpretable long short-term memory networks for crop yield estimation. IEEE Geosci. Remote Sens. Lett. 20, 1–5. doi: 10.1109/LGRS.2023.3244064, PMID: 41116384

[B103] Mehdipour S. Mirroshandel S. A. Tabatabaei S. A. (2025). Vision transformers in precision agriculture: A comprehensive survey. Intelligent Syst. Appl., 29, 200617. doi: 10.1016/j.iswa.2025.200617, PMID: 41834798

[B104] Morales G. Sheppard J. W. Hegedus P. B. Maxwell B. D. (2023). Improved yield prediction of winter wheat using a novel two-dimensional deep regression neural network trained via remote sensing. Sensors 23, 489. doi: 10.3390/s23010489, PMID: 36617083 PMC9824857

[B105] Morales A. Villalobos F. J. (2023). Using machine learning for crop yield prediction in the past or the future. Front. Plant Sci. 14, 1128388. doi: 10.3389/fpls.2023.1128388, PMID: 37063228 PMC10097960

[B106] Mowla M. N. Mowla N. Shah A. S. Rabie K. M. Shongwe T. (2023). Internet of Things and wireless sensor networks for smart agriculture applications: A survey. IEEE Access 11, 145813–145852. doi: 10.1109/ACCESS.2023.3346299, PMID: 41116384

[B107] Muhammad A. Khan Z. U. Khan J. Mashori A. S. Ali A. Jabeen N. . (2025). A comprehensive review of crop stress detection: destructive, non-destructive, and ML-based approaches. Front. Plant Sci. 16, 1638675. doi: 10.3389/fpls.2025.1638675, PMID: 40978773 PMC12447170

[B108] Mustak S. Uday G. Ramesh B. Praveen B. (2019). Evaluation of the performance of SAR and SAR-optical fused dataset for crop discrimination. Int. Arch. Photogrammetry Remote Sens. Spatial Inf. Sci. 42, 563–571. doi: 10.5194/isprs-archives-XLII-3-W6-563-2019, PMID: 38859159

[B109] Naik H. R. Amin T. Sheraz Mahdi S. (2022). “ Post-harvest management and value addition of food crops,” in Secondary agriculture: sustainability and livelihood in India ( Springer), London (global), Berlin (corporate), New York (sales), 131–146.

[B110] Natraj N. Raj P. Karpagam M. Gunanandhini S. (2025). A study on EDGE AI application in crop monitoring. Edge Intelligence: Exploring Front. AI at Edge 51-72. doi: 10.1002/9781394314409.ch2, PMID: 41823357

[B111] Nejad S. M. M. Abbasi-Moghadam D. Sharifi A. Farmonov N. Amankulova K. Lászlź M. (2022). Multispectral crop yield prediction using 3D-convolutional neural networks and attention convolutional LSTM approaches. IEEE J. Selected Topics Appl. Earth Observations Remote Sens. 16, 254–266. doi: 10.1109/JSTARS.2022.3223423, PMID: 41116384

[B112] Orduna-Cabrera F. Rios-Ochoa A. Frank F. Lindner S. Sandoval-Gastelum M. Obersteiner M. . (2025). Short-term forecasting arabica coffee cherry yields by seq2Seq over LSTM for smallholder farmers. Sustainability 17, 3888. doi: 10.3390/su17093888, PMID: 41725453

[B113] Pei J. Tan S. Zou Y. Liao C. He Y. Wang J. . (2025). The role of phenology in crop yield prediction: Comparison of ground-based phenology and remotely sensed phenology. Agric. For. Meteorol 361, 110340. doi: 10.1016/j.agrformet.2024.110340, PMID: 41834798

[B114] Piekutowska M. Niedbała G. Piskier T. Lenartowicz T. Pilarski K. Wojciechowski T. . (2021). The application of multiple linear regression and artificial neural network models for yield prediction of very early potato cultivars before harvest. Agronomy 11, 885. doi: 10.3390/agronomy11050885, PMID: 41725453

[B115] Pirzada T. Affokpon A. Guenther R. H. Mathew R. Agate S. Blevins A. . (2023). Plant-biomass-based hybrid seed wraps mitigate yield and post-harvest losses among smallholder farmers in sub-Saharan Africa. Nat. Food 4, 148–159. doi: 10.1038/s43016-023-00695-z, PMID: 37117858 PMC10154224

[B116] Prasad N. Patel N. Danodia A. (2021). Crop yield prediction in cotton for regional level using random forest approach. Spatial Inf. Res. 29, 195–206. doi: 10.1007/s41324-020-00346-6, PMID: 41835834

[B117] Prey L. Hanemann A. Ramgraber L. Seidl-Schulz J. Noack P. O. (2022). UAV-Based estimation of grain yield for plant breeding: Applied strategies for optimizing the use of sensors, vegetation indices, growth stages, and machine learning algorithms. Remote Sens. 14, 6345. doi: 10.3390/rs14246345, PMID: 41725453

[B118] Proctor J. (2021). Atmospheric opacity has a nonlinear effect on global crop yields. Nat. Food 2, 166–173. doi: 10.1038/s43016-021-00240-w, PMID: 37117447

[B119] Qiao M. He X. Cheng X. Li P. Luo H. Zhang L. . (2021). Crop yield prediction from multi-spectral, multi-temporal remotely sensed imagery using recurrent 3D convolutional neural networks. Int. J. Appl. Earth Observation Geoinformation 102, 102436. doi: 10.1016/j.jag.2021.102436, PMID: 41834798

[B120] Rahman H. Imran H. M. Hossain A. Siddiqui M. I. H. Haque A. (2025). Explainable vision transformers for real−time chili and onion leaf disease identification and diagnosis. Int. J. Sci. Res. Arch., 15, 1823–1833. doi: 10.30574/ijsra.2025.15.1.1163

[B121] Rajendiran G. Rethnaraj J. (2025). Lettuce yield prediction: elasticNet regression model (ElNetRM) for indoor aeroponic vertical farming system. Int. J. Electrical Comput. Eng. Syst. 16, 683–695. doi: 10.32985/ijeces.16.9.5

[B122] Rashid M. Bari B. S. Yusup Y. Kamaruddin M. A. Khan N. (2021). A comprehensive review of crop yield prediction using machine learning approaches with special emphasis on palm oil yield prediction. IEEE Access 9, 63406–63439. doi: 10.1109/ACCESS.2021.3075159, PMID: 41116384

[B123] Raza I. Zubair M. Zaib M. Khalil M. H. Haidar A. Sikandar A. . (2023). Precision nutrient application techniques to improve soil fertility and crop yield: A review with future prospect. Int. Res. J. Educ. Tecnology 5.

[B124] Reddy D. J. Madapuri R. K. (2024). Agriculture crop yield prediction using inertia based cat swarm optimization. Int. J. Electrical Comput. Eng. 14, 1700–1710. doi: 10.11591/ijece.v14i2.pp1700-1710

[B125] Rivera G. Porras R. Florencia R. Sánchez-Solís J. P. (2023). LiDAR applications in precision agriculture for cultivating crops: A review of recent advances. Comput. Electron. Agric. 207, 107737. doi: 10.1016/j.compag.2023.107737, PMID: 41834798

[B126] Sadenova M. A. Beisekenov N. A. Rakhymberdina M. Y. Varbanov P. S. Klemeš J. J. (2021). Mathematical modelling in crop production to predict crop yields. Chem. Eng. Trans. 88, 1225–1230. doi: 10.3303/CET2188204

[B127] Sakamoto T. Gitelson A. A. Arkebauer T. J. (2013). MODIS-based corn grain yield estimation model incorporating crop phenology information. Remote Sens. Environ. 131, 215–231. doi: 10.1016/j.rse.2012.12.017, PMID: 41834798

[B128] Saki M. Keshavarz R. Franklin D. Abolhasan M. Lipman J. Shariati N. (2025). A data-driven review of remote sensing-based data fusion in precision agriculture from foundational to transformer-based techniques. IEEE Access. 13, 166188–166209. doi: 10.1109/ACCESS.2025.3610649, PMID: 41116384

[B129] Sakthipriya S. Naresh R. (2022). Effective energy estimation technique to classify the nitrogen and temperature for crop yield based green house application. Sustain. Computing: Inf. Syst. 35, 100687. doi: 10.1016/j.suscom.2022.100687, PMID: 41834798

[B130] Salomi M. Kiruthika J. Samsudeen S. Maragatham G. (2025). “ Crop yield estimation,” in Artificial intelligence and computer vision for ecological informatics ( CRC Press), Milton Park, Abingdon-on-Thames in the United Kingdom, 294–304.

[B131] Setiya P. Nain A. S. Satpathi A. (2024). Comparative analysis of SMLR, ANN, Elastic net and LASSO based models for rice crop yield prediction in Uttarakhand. Mausam 75, 191–196. doi: 10.54302/mausam.v75i1.3576

[B132] Shafi U. Mumtaz R. Anwar Z. Ajmal M. M. Khan M. A. Mahmood Z. . (2023). Tackling food insecurity using remote sensing and machine learning-based crop yield prediction. IEEE Access 11, 108640–108657. doi: 10.1109/ACCESS.2023.3321020, PMID: 41116384

[B133] Shafiee S. Lied L. M. Burud I. Dieseth J. A. Alsheikh M. Lillemo M. (2021). Sequential forward selection and support vector regression in comparison to LASSO regression for spring wheat yield prediction based on UAV imagery. Comput. Electron. Agric. 183, 106036. doi: 10.1016/j.compag.2021.106036, PMID: 41834798

[B134] Shafiee S. Mroz T. Burud I. Lillemo M. (2023). Evaluation of UAV multispectral cameras for yield and biomass prediction in wheat under different sun elevation angles and phenological stages. Comput. Electron. Agric. 210, 107874. doi: 10.1016/j.compag.2023.107874, PMID: 41834798

[B135] Shaikh M. S. Jha A. K. Soni B. R. Patel R. N. K. PM D. (2025). “ Flying edge intelligence: UAV-driven edge computing for autonomous precision farming,” in 2025 international conference on emerging technologies in engineering applications (ICETEA) ( IEEE), New York City, USA, 1–6.

[B136] Shee A. Parmar A. Raut S. Strum B. Bennett B. (2023). Assessing the measurement methods of post-harvest food loss and waste: opportunities and challenges. Enterprise Dev. Microfinance 33, 1–16. doi: 10.3362/1755-1986.22-00062

[B137] Shen Y. Mercatoris B. Cao Z. Kwan P. Guo L. Yao H. . (2022a). Improving wheat yield prediction accuracy using LSTM-RF framework based on UAV thermal infrared and multispectral imagery. Agriculture 12, 892. doi: 10.3390/agriculture12060892, PMID: 41725453

[B138] Shen Y. Wang L. Jin Y. (2022). “ AAFormer: A multi-modal transformer network for aerial agricultural images,” in Proceedings of the IEEE/CVF conference on computer vision and pattern recognition, IEEE, New York City, U.S. 1705–1711.

[B139] Shuai G. Basso B. (2022). Subfield maize yield prediction improves when in-season crop water deficit is included in remote sensing imagery-based models. Remote Sens. Environ. 272, 112938. doi: 10.1016/j.rse.2022.112938, PMID: 41834798

[B140] Sillmann J. Aunan K. Emberson L. Büker P. Van Oort B. O’Neill C. . (2021). Combined impacts of climate and air pollution on human health and agricultural productivity. Environ. Res. Lett. 16, 093004. doi: 10.1088/1748-9326/ac1df8

[B141] Singh J. Biswas B. Dhaliwal L. K. Poddar R. Gharib A. F. Raafat B. M. . (2025). Performance evaluation of the DSSAT-CERES-Wheat and WOFOST-Wheat models under various agroclimatic conditions in northwest India. Theor. Appl. Climatol 156, 1–18. doi: 10.1007/s00704-025-05476-1, PMID: 41835834

[B142] Sisheber B. Marshall M. Mengistu D. Nelson A. (2023). Assimilation of Earth observation data for crop yield estimation in smallholder agricultural systems. IEEE J. Selected Topics Appl. Earth Observations Remote Sens. 17, 557–572. doi: 10.1109/JSTARS.2023.3329237, PMID: 41116384

[B143] Sivakumar M. (2023). Importance of solar radiation and the need for improved respect to Sun by Agrometeorologists. J. Agrometeorol 25, 51–60. doi: 10.54386/jam.v25i1.1971

[B144] Son N. Chen C. Chen C. Minh V. Trung N. (2014). A comparative analysis of multitemporal MODIS EVI and NDVI data for large-scale rice yield estimation. Agric. For. meteorol 197, 52–64. doi: 10.1016/j.agrformet.2014.06.007, PMID: 41834798

[B145] Sontowski S. Gupta M. Chukkapalli S. S. L. Abdelsalam M. Mittal S. Joshi A. . (2020). “ Cyber attacks on smart farming infrastructure,” in 2020 IEEE 6th international conference on collaboration and internet computing (CIC) ( IEEE). New York City, USA.

[B146] SP P. Balamurugan P. (2022). “ Unmanned aerial vehicle in the smart farming systems: Types, applications and cyber-security threats,” in 2022 international conference on innovative computing, intelligent communication and smart electrical systems (ICSES) ( IEEE), New York City, USA, 1–9.

[B147] Srivastav A. K. Das P. (2025). “ Edge computing and AI in agricultural ioT,” in Biotechnology and ioT in agriculture and food production: green innovation ( Springer), London (global), Berlin (corporate), New York (sales), 187–200.

[B148] Sudhamathi T. Perumal K. (2024). Ensemble regression based Extra Tree Regressor for hybrid crop yield prediction system. Measurement: Sensors 35, 101277. doi: 10.1016/j.measen.2024.101277, PMID: 41834798

[B149] Sunoj S. Cho J. Guinness J. van Aardt J. Czymmek K. J. Ketterings Q. M. (2021). Corn grain yield prediction and mapping from Unmanned Aerial System (UAS) multispectral imagery. Remote Sens. 13, 3948. doi: 10.3390/rs13193948, PMID: 41725453

[B150] Taylor S. D. Browning D. M. (2022). Classification of daily crop phenology in phenocams using deep learning and hidden markov models. Remote Sens. 14, 286. doi: 10.3390/rs14020286, PMID: 41725453

[B151] Tripathi A. Tiwari R. K. Tiwari S. P. (2022). A deep learning multi-layer perceptron and remote sensing approach for soil health based crop yield estimation. Int. J. Appl. Earth Observation Geoinformation 113, 102959. doi: 10.1016/j.jag.2022.102959, PMID: 41834798

[B152] Tunio M. H. Ping Li J. Zeng X. Ahmed A. Shah S. A. Shaikh H.-U. . (2024). Advancing plant disease classification: A robust and generalized approach with transformer-fused convolution and Wasserstein domain adaptation. Comput. Electron. Agric. 227, 109574. doi: 10.1016/j.compag.2024.109574, PMID: 41834798

[B153] Uribeetxebarria A. Castellón A. Aizpurua A. (2023). Optimizing wheat yield prediction integrating data from Sentinel-1 and Sentinel-2 with CatBoost algorithm. Remote Sens. 15, 1640. doi: 10.3390/rs15061640, PMID: 41725453

[B154] Vaidya R. Nalavade D. Kale K. (2022). Hyperspectral imagery for crop yield estimation in precision agriculture using machine learning approaches: A review. Int. J. Creat Res. Thoughts 9, a777–a789.

[B155] Valente D. S. M. Pereira G. W. de Queiroz D. M. Zandonadi R. S. do Amaral L. R. Bottega E. L. . (2024). Accuracy of various sampling techniques for precision agriculture: A case study in Brazil. Agriculture mdpi 14, 2198. doi: 10.3390/agriculture14122198, PMID: 41725453

[B156] Vallentin C. Harfenmeister K. Itzerott S. Kleinschmit B. Conrad C. Spengler D. (2022). Suitability of satellite remote sensing data for yield estimation in northeast Germany. Precis. Agric. 23, 52–82. doi: 10.1007/s11119-021-09827-6, PMID: 41835834

[B157] Van Der Linden D. Michalec O. A. Zamansky A. (2020). Cybersecurity for smart farming: socio-cultural context matters. IEEE Technol. Soc. Magazine 39, 28–35. doi: 10.1109/MTS.2020.3031844, PMID: 41116384

[B158] Van Wart J. Kersebaum K. C. Peng S. Milner M. Cassman K. G. (2013). Estimating crop yield potential at regional to national scales. Field Crops Res. 143, 34–43. doi: 10.1016/j.fcr.2012.11.018, PMID: 41834798

[B159] Vashisht S. Kumar P. Trivedi M. C. (2023). Crop yield prediction using improved extreme learning machine. Commun. Soil Sci. Plant Anal. 54, 1–21. doi: 10.1080/00103624.2022.2108828, PMID: 41799851

[B160] Wang Y. Wang P. Tansey K. Liu J. Delaney B. Quan W. (2025). An interpretable approach combining Shapley additive explanations and LightGBM based on data augmentation for improving wheat yield estimates. Comput. Electron. Agric. 229, 109758. doi: 10.1016/j.compag.2024.109758, PMID: 41834798

[B161] Wu Y. Al-Jumaili S. J. Al-Jumeily D. Bian H. (2022). Prediction of the nitrogen content of rice leaf using multi-spectral images based on hybrid radial basis function neural network and partial least-squares regression. Sensors 22, 8626. doi: 10.3390/s22228626, PMID: 36433222 PMC9695716

[B162] Wu B. Fan L. Xu B. Yang J. Zhao R. Wang Q. . (2025). UAV-based LiDAR and multispectral sensors fusion for cotton yield estimation: Plant height and leaf chlorophyll content as a bridge linking remote sensing data to yield. Ind. Crops Prod 230, 121110. doi: 10.1016/j.indcrop.2025.121110, PMID: 41834798

[B163] Yadav R. Seth A. Dembla N. (2024). Optimizing crop yield prediction: data-driven analysis & Machine learning modeling using USDA datasets. Curr. Agric. Res. J. 12, 272–285. doi: 10.12944/CARJ.12.1.22

[B164] Yan Y. Wang Y. Li J. Zhang J. Mo X. (2025). Crop yield time-series data prediction based on multiple hybrid machine learning models. Appl. Comput. Eng. Учредители: EWA Publishing 133, 217–233. doi: 10.48550/arXiv.2502.10405, PMID:

[B165] Yang W. Nigon T. Hao Z. Paiao G. D. Fernández F. G. Mulla D. . (2021). Estimation of corn yield based on hyperspectral imagery and convolutional neural network. Comput. Electron. Agric. 184, 106092. doi: 10.1016/j.compag.2021.106092, PMID: 41834798

[B166] Yuan J. Zhang Y. Zheng Z. Yao W. Wang W. Guo L. (2024). Grain crop yield prediction using machine learning based on UAV remote sensing: A systematic literature review. Drones 8, 559. doi: 10.3390/drones8100559, PMID: 41725453

[B167] Zangana H. M. Luckyardi S. Mustafa F. M. Li S. (2025). “ Enhancing agricultural cybersecurity: leveraging deep learning and large language models for smart farming protection,” in Revolutionizing cybersecurity with deep learning and large language models ( IGI Global Scientific Publishing), Hershey, Pennsylvania, USA, 307–338.

[B168] Zeng L. Peng G. Meng R. Man J. Li W. Xu B. . (2021). Wheat yield prediction based on unmanned aerial vehicles-collected red–green–blue imagery. Remote Sens. 13, 2937. doi: 10.3390/rs13152937, PMID: 41725453

[B169] Zhang M. Chen T. E. Gu X. Chen D. Wang C. Wu W. . (2023). Hyperspectral remote sensing for tobacco quality estimation, yield prediction, and stress detection: A review of applications and methods. Front. Plant Sci. 14, 1073346. doi: 10.3389/fpls.2023.1073346, PMID: 36968402 PMC10030857

[B170] Zhang X. Han L. Sobeih T. Lappin L. Lee M. A. Howard A. . (2022). The self-supervised spectral–spatial vision transformer network for accurate prediction of wheat nitrogen status from UAV imagery. Remote Sens. 14, 1400. doi: 10.3390/rs14061400, PMID: 41725453

[B171] Zhang M. Xu Z. Wang P. Li R. Wang L. Liu Q. . (2025). AgriDoctor: A multimodal intelligent assistant for agriculture. arXiv preprint arXiv:.17044. doi: 10.48550/arXiv.2509.17044, PMID: 41363103

[B172] Zhao J. Sun D. Mi J. Zhao K. Peng J. Tu K. . (2025). Hyperspectral imaging coupled with transformer enhanced convolutional autoencoder architecture towards real-time multi-target classification of damaged soybeans. Food Control 111606. doi: 10.1016/j.foodcont.2025.111606, PMID: 41834798

[B173] Zhu X. Nie S. Wang C. Xi X. Hu Z. (2018). A ground elevation and vegetation height retrieval algorithm using micro-pulse photon-counting lidar data. Remote Sens. 10, 1962. doi: 10.3390/rs10121962, PMID: 41725453

